# Insulin-like growth factor 5 associates with human Aß plaques and promotes cognitive impairment

**DOI:** 10.1186/s40478-022-01352-5

**Published:** 2022-05-05

**Authors:** Stefanie Rauskolb, Thomas Andreska, Sophie Fries, Cora Ruedt von Collenberg, Robert Blum, Camelia-Maria Monoranu, Carmen Villmann, Michael Sendtner

**Affiliations:** 1 Institute of Clinical Neurobiology, University of Würzburg, Versbacher-Str. 5, 97078 Würzburg, Germany; 2 Department of Neuropathology, Institute of Pathology, University of Würzburg, Josef-Schneider-Str. 2, 97080 Würzburg, Germany; 3 Department of Neurology, University Hospital Würzburg, Josef-Schneider-Str. 11, 97080 Würzburg, Germany

**Keywords:** Igfbp5, Alzheimer, Bdnf and exercise

## Abstract

Risk factors such as dysregulation of Insulin-like growth factor (IGF) signaling have been linked to Alzheimer’s disease. Here we show that Insulin-like Growth Factor Binding Protein 5 (Igfbp5), an inhibitory binding protein for insulin-like growth factor 1 (Igf-1) accumulates in hippocampal pyramidal neurons and in amyloid plaques in brains of Alzheimer patients. We investigated the pathogenic relevance of this finding with transgenic mice overexpressing Igfbp5 in pyramidal neurons of the brain. Neuronal overexpression of Igfbp5 prevents the training-induced increase of hippocampal and cortical Bdnf expression and reduces the effects of exercise on memory retention, but not on learning acquisition. Hence, elevated IGFBP5 expression could be responsible for some of the early cognitive deficits that occur during the course of Alzheimer’s disease.

**Supplementary Information:**

The online version contains supplementary material available at 10.1186/s40478-022-01352-5.

## Introduction

The beneficial effects of physical activity on the nervous system are numerous and include a wide range of interrelated effects on brain structure, brain function, and cognition [24, 25, 31, 57]. These beneficial effects primarily involve the hippocampus, which is known to be critical for many cognitive functions which are severely affected in Alzheimer’s disease [77]. Adaptations observed in the hippocampus after exercise include increased neurogenesis and synaptic plasticity [22]. A known mediator of these exercise-induced plastic changes is Brain-derived neurotrophic factor (Bdnf) [5]. The expression of this factor is increased after exercise, particularly in the hippocampus and cortex [23, 52]. Bdnf is known for its essential function in synaptic plasticity, learning and memory [10, 38, 55, 83]. Though these results are widely recognized, the underlying molecular mechanisms that link Bdnf expression and physical activity are still not fully understood [34, 70, 80]. Previous studies have shown that Bdnf and Insulin-like growth factor 1 (Igf-1) signaling pathways act together in mediating the effects of physical exercise, as the blockade of their hippocampal receptors, TrkB or Igf-1r, limits the exercise-induced improvement of spatial learning tasks, as well as the exercise-induced expression of synaptic proteins [19, 26, 30, 39, 49, 73, 74]. The bioavailability and bioactivity of Igf-1 in the mammalian brain is controlled by a family of seven high affinity insulin-like growth factor binding proteins (Igfbps) [2, 12, 32, 61, 64]. These Igfbps could play a role in memory formation, and they are also involved in Alzheimer pathogenesis [1, 33, 65]. Recently, Igfbp5 was found to be upregulated in the hippocampus and cortex of an Alzheimer's disease mouse model and in the cerebrospinal fluid of Alzheimer's disease cases [8, 65], suggesting a pathogenic role of IGFBP5 in this disease. In the present work, we investigated the expression of IGFBP5 in the hippocampus of Alzheimer’s disease postmortem brain and tested in mice the effects of elevated Igfbp5 expression on hippocampal Bdnf protein levels and whether Igfbp5 might impair with the effects of exercise on cognition. Our analyses revealed a strong effect of elevated Igfbp5 on memory performance that correlated with impaired induction of exercise induced Bdnf expression.

## Material and methods

### Human brain tissue and immunohistochemistry

Human brain tissues from thirteen cases diagnosed with Alzheimer disease (AD) according to neuropathological Braak [15] and CERAD criteria [36, 50] and four control cases (postmortem delay between 17 and 36 h; age between 62 and 86 years at time of death) were obtained from the brain bank at the Department of Neuropathology at Würzburg University Hospital and used for research purposes as approved by the local Ethics Committee of the Faculty of Medicine at the University of Würzburg (78/99; 99/11). All patients and controls, or their next of kin, had given informed consent for autopsy and use of their brain tissue for research purposes. Clinical data and other variables from patients were obtained from the caring clinician of each patient (Table 1). For immunohistochemistry, frontal and temporal lobe sections including temporal isocortex, hippocampus and enthorinal cortex were used. 3,3′-diaminobenzidine tetrahydrochloride hydrate (DAB)- and 3-Amino-9-Ethylcarbazole (AEC)- developed immunohistochemical single stainings were performed on deparaffinized and rehydrated formalin-fixed paraffin-embedded human brain tissue sections (3–8 μm). Antigen retrieval was performed for 10 min by steaming of tissue sections in 20 mM citrate buffer (pH 6.0) for IGFBP5 or in 80% formic acid for 60 min at room temperature for Amyloid beta 42 (Aß42) and paired helical filament (PHF)-tau (AT8). Endogenous peroxidase activity was blocked with 0.7% hydrogen peroxide (AppliChem) for 15 min at room temperature. Blocking of non-specific interactions of the primary antibodies was achieved in a solution of 10% normal goat or rabbit serum (Life technologies) for 20 min at room temperature. Primary antibodies (IGFBP5 (R&D Systems, Cat# AF875, RRID:AB_355678); non-specific goat immunoglobulin G (IgG) (R&D Systems, Cat# AB-108-C, RRID:AB_354267); Aß42 (Covance, Cat# SIG-39220, RRID:AB_662812**)**; PHF-Tau (Thermo Fisher Scientific, Cat# MN1020, RRID:AB_223647); TDP-43 (Proteintech, Cat# 10782-2-AP, RRID:AB_615042) were incubated overnight at 4 °C, except for TDP-43, which was incubated at room temperature for 40 min. Biotinylated secondary antibodies and peroxidase-conjugated streptavidin were each applied for 30 min at room temperature (DCS, # PD000POL, Biogenex, # LP000-ULE). Thereafter, human brain sections were developed with 3,3′-diaminobenzidine (DAB, Dako, K3468) or with 3-Amino-9-Ethylcarbazole (AEC, Romulin AEC Chromogen Kit, RAEC810L) for 7–10 min. Human brain sections were counterstained with hemalaun (Carl Roth, Cat #3816.2) to stain the cell nuclei blue, dehydrated in ethanol, cleared with xylene, and mounted with Pertex (Biosystems, Cat #41-4011-00). Images were acquired under standardized conditions by controlling the lamp brightness (Zeiss, Axioplan2).

**Table 1:** Summary of clinical and pathological data

Case	Gender	Age (years)	Brain weight (g)	Demented	Braak stage [15]	CERAD stage [36, 50]	Hippocampal TDP43 pathology [44, 53]	Other pathology
1	Female	71	1050	No	0	0	No	No
2	Male	66	1350	No	0	0	No	No
3	Male	69	n.a	No	0	0	No	No
4	Male	62	1350	No	0	0	No	No
5	Male	69	1550	No	I	A	No	No
6	Female	86	1000	Yes	II	0	No	Diabetes, Argyrophilic grain disease
7	Female	77	1200	No	III	A	No	No
8	Male	64	1160	No	III	A	No	Argyrophilic grain disease
9	Female	85	n.a	n.a	III	A	No	No
10	Female	86	1040	Yes	III	B	No	No
11	Female	77	1116	No	IV	B	No	No
12	Female	74	960	Yes	V	C	No	No
13	Female	85	1020	Yes	V	C	No	No
14	Male	76	1350	Yes	V	C	No	No
15	Female	83	1050	Yes	VI	C	Yes	No
16	Female	80	930	Yes	VI	C	No	Korsakow syndrom
17	Male	71	1200	Yes	VI	C	No	No

*IGFBP5—Ubiquitin Double Immunohistochemistry* To determine co-localization of IGFBP5 with ubiquitin, sections were deparaffinized in xylene and rehydrated in a descending ethanol series (100%, 96%, 70%). Epitope retrieval was achieved by heating the slides in 20 mM citrate buffer (pH 6.0) for 10 min in a high-pressure cooker. After cooling down to room temperature and 2 washing steps for 2 min in double distilled water, an incubation step in 0.7% hydrogen peroxide (AppliChem) for 15 min was included to ensure blockage of endogenous peroxidase. Sections were subjected to the primary rabbit anti-Ubiquitin antibody (Zytomed Systems, Cat # 521-3350, RRID: AB_2864643) diluted 1:100 in antibody diluent (Zytomed Systems, Cat # ZU025-100) for 40 min at room temperature. After three washing steps for 5 min in 1× TBS (pH 7.4) sections were incubated with the horseradish peroxidase (HRP)-coupled polymer from the ZytoChem-Plus HRP-Polymer-Kit (Zytomed Systems, Cat # ZUC032-006, RRID: AB_2868566) for 30 min. After three washing steps for 5 min in 1× TBS (pH 7.4), 3,3′-diaminobenzidine (DAB) (Zytomed Systems, Cat # DAB 530) was applied to the sections for 7 min for colour development. Subsequently, sections were washed three times in 1× TBS (pH 7.4) for 5 min and subjected to the primary goat anti-IGFBP5 antibody (R&D Systems, Cat# AF875, RRID: AB_355678) diluted 1:400 in 1× TBS/3% BSA (Fraction V, AppliChem, #A13910100) overnight at 4 °C. Following another 3 washing steps in 1× TBS (pH 7.4) for 5 min, bound antibody was detected using Goat-on-Rodent AP-Polymer (Biocare Medical, Cat # GAP514G) employing Permanent AP Red Kit (Zytomed Systems, Cat # ZUC001-125) as substrate. Following counterstaining with hemalaun (Carl Roth GmbH Cat # 3816) to stain the cell nuclei blue, human sections were dehydrated in ethanol, cleared with xylene, and mounted with Pertex (VWR, Cat # LEIC801). Images were acquired under standardized conditions by controlling the lamp brightness (Zeiss, Axioplan2).

### Mice

*Igfbp-5* transgenic mice (*Bp5* tg+) were generated on C57BL/6 background as described previously [68, 69] (further information on these mice see Additional file 1: Supplementary Material Section). All experiments were approved by a license for animal testing (RUF-55.2.-2532.-2-530-10) and performed in accordance with the supervision through local veterinary authority (Veterinaeramt der Stadt Wuerzburg) and Committee on the Ethics of Animal Experiments (i.e., Regierung von Unterfranken Wuerzburg). For biochemical and immunohistochemical experiments, both male and female mice were used, whereas for behavioral experiments, only male mice were used and housed individually under a 12 h light/dark cycle (6:00 a.m.–6:00 p.m.) with ad libitum access to food and water. Mice were allowed to habituate to a new housing facility at least one week prior to the start of the experiment. The cages (Tecniplast, 1264 C Eurostandard Typ II, 365 × 207 × 140 mm) were kept in a Scantainer (Scanbur Ltd. Denmark) assuring stable conditions through a constant airflow and maintaining a temperature of about 21 °C and air humidity of about 55%. Experimental procedures took place during the light phase of the cycle between 7:00 am and 5:00 pm. Mice were transported in their home cage to a separate room and allowed to calm down for half an hour. Then mice were taken individually in their home cages to the experimental room for experiments. The experimenter was unaware of the genotype to keep experiments unbiased. The age of mice used for individual experiments is indicated accordingly throughout the results section.

### Behaviour

*Voluntary wheel running*. Mice were individually housed for 7 days with (runners) or without (sedentary) continuous access to a voluntary running wheel (Tecniplast, model #1284L0106). The mouse cage measured 365 × 207 × 140 mm with a wheel diameter of 23 cm. Each running wheel was equipped with a magnetic switch and LCD counter to record number of revolutions. The rotations of the running wheel and corresponding daily running distances (km) were recorded daily.

*Open field test.* Mice were placed in a 48 × 48 × 50 cm square box, illuminated with ∼40 lx (Light meter, Voltcraft MS1300). Animals were monitored for 10 min each and tracked with the Video Mot Software (TSE Systems, Bad Homburg, Germany, RRID: SCR_014334). For analysis, the box was divided into fields of interest: center of the arena (24 × 24 cm) versus the periphery and the entries into the centre, time spent in the center and traveled distances were recorded by one-point body tracking.

*Y-Maze Test* To determine spontaneous alternation mice were placed at the end of the start arm, facing the center, and allowed to explore the maze freely for 8 min (reference run). The sequence of arm entries and the total number of arm entries was recorded. Alternation behaviour was defined as a complete cycle of consecutive entrances into each of the 3 arms without repetition (e.g., the sequence, ABCBCBCA was counted as two alternations, with the first consecutive ABC and the last consecutive BCA out of six consecutive occasions). The dependent variables were activity, defined as the number of arms entered, and percent alternation, calculated as the number of alternations (entries into three different arms consecutively) divided by the total possible alternations (i.e., the number of arms entered minus 2) and multiplied by 100. To determine spatial reference memory, mice were placed into one of the arms of the maze (start arm) and allowed to explore the maze with one of the arms closed for 10 min (training trial). After a 1-h intertrial interval, mice were returned to the Y maze by placing them in the start arm. Then, the mice were allowed to explore freely all three arms of the maze for 5 min (test trial). The number of entries into and the time spent in each arm were registered from video recordings by an observer blind to the genotype of the mice. All sessions were video recorded through a camera (Logitech) mounted above the maze and mouse position was tracked using the Video Mot Software (TSE Systems, Bad Homburg, Germany, RRID:SCR_014334). Room illumination was kept at 40 lx (Light meter, Voltcraft MS1300).

*Morris water maze test.* Mice were trained in the Morris water maze [51, 76] to find a hidden platform 1 cm below the surface of the pool (1.40-m diameter) filled with water (23 °C). The training of mice was always performed by the same person each day. Stark visual cues were placed around the room containing the pool. Within the pool, a 10 cm circular Plexiglas escape platform was submerged in one of the quadrants 1 cm below the water's surface. For training, mice were placed into the pool in one of four starting locations (north, south, east, or west), and allowed to swim until they located the escape platform (corresponds to the target area in the probe trial on day 6). Upon finding the platform, mice were permitted to rest for 10 s before being removed. If they did not locate the platform within 60 s, mice were guided manually to the platform and given 10 s of rest before being removed. Mice received four training trials per day for five days and were placed in temporary cages between the training trials of a particular day. Upon completion of training, the platform was removed 24 h after the last training session (day 6) and mice were allowed to swim for 60 s in the pool. The percentage of time and the distance spent in the platform area, which is a small part of the target quadrant was calculated for each animal. For more information on the calculation of time in the platform area see Additional file 1: Supplementary Material Section. Latency to find the platform, swim speed, distance and time spent in the platform area were recorded semi-automatically by a video tracking system (Video Mot Software, TSE Systems, Bad Homburg, Germany, RRID:SCR_014334).

### Enzyme-linked immunosorbent assay (ELISA)

Bdnf quantification was performed by ELISA as previously described [46, 62]. The chemiluminescence was measured with the plate reader Tecan Infinite M200 PRO (RRID:SCR_019033).

### Immunohistochemistry

*Igfbp-5 immunohistochemistry* Mice were euthanized by an overdose of CO_2_ and transcardially perfused with 4% paraformaldehyde (Carl Roth, Cat #0335.3, PFA) in 0.1 M phosphate buffer, pH 7.4. Brains were dissected, post-fixed in 4% PFA for 3 h, transferred to phosphate-buffered saline and embedded in paraffin. 5µm thick serial sections were deparaffinized with xylene and dehydrated through a graded ethanol series (100%, 96%, 70%). Antigen retrieval was accomplished by incubating the sections in 20 mM citrate buffer (pH 6) for 10 min in a household pressure cooker. Igfbp5 (R&D Systems, Cat #AF875, RRID: AB_355678) immunoreactivity was visualized with the anti-goat-HRP-DAB Cell Tissue Staining Kit (R&D Systems, Cat # CTS008, RRID: AB_10052005) according to the manufacturer’s instructions and sections were counterstained with hemalaun. Negative controls were prepared using non-specific goat immunoglobulin G (IgG) (R&D Systems, Cat # AB-108-C, RRID: AB_354267) in place of primary antibodies. Images were acquired under standardized conditions by controlling the lamp brightness (Zeiss, Axioplan2).

*Bdnf, Synaptophysin immunohistochemistry* Mice were deeply anesthetized with 120 mg/kg ketamine hydrochloride and 16 mg/kg xylazine hydrochloride in 0.4–0.6 ml 1× PBS and transcardially perfused with 0.4% heparin in 1× PBS for 2–3 min followed by an eight-minute perfusion with 2% PFA in 0.1 M phosphate buffer, pH 6.0. Brains were removed and postfixed with 2–4% PFA in 0.1 M phosphate buffer at 4 °C for 0.5–2 h before thorough washing in 1 × PBS and embedded in 4% agarose. Coronal brain sections (20–40 μm) were cut on a vibratome (Leica Microsystems, VT1000S, RRID:SCR_016495) and collected and stored in Cryoprotection buffer (1× PBS, glycerol, ethylene glycol) at − 20 °C. Presynaptic terminals were detected with guinea pig polyclonal antibodies against synaptophysin (Synaptic Systems Cat# 101004, RRID:AB_1210382). Bdnf was detected with mouse monoclonal anti-Bdnf antibodies (DSHB Cat# Bdnf-#9, RRID:AB_2617199) [28, 46]. For staining sections were washed in 1× PBS followed by permeabilization and blocked with 0.3% Triton-X-100, 0.1% Tween 20, and 10% normal donkey serum (Bio-Rad Cat# C06SB) in 1× PBS for 2 h. Primary antibodies were diluted in permeabilization and blocking buffer and incubated at a final concentration between 0.4 and 1.0 µg/ml in the presence of 0.01% NaN_3_ on a shaker at 4 °C for 72 h. Brain sections were extensively washed in 1× PBS, 0.1% Triton-X-100, 0.3% Tween20. For fluorescent signal detection, the following secondary antibodies were diluted in permeabilization and blocking buffer at a final concentration of 0.625 µg/ml: donkey anti Mouse DyLight549 (Jackson Immunoresearch; 715-505-150), Donkey anti Mouse DyLight550 (Thermo Fisher Scientific; #SA5-10167, RRID: AB_2556747) and donkey anti guinea pig Cy5 (Jackson ImmunoResearch Labs, Cat# 706-175-148, RRID:AB_2340462). Nuclei were stained with 0.4 µg/ml DAPI (Thermo Fisher Scientific, Cat# D1306, RRID: AB_2629482). Labeled sections were mounted onto glass slides (Thermo Fisher Scientific, #J1800AMNZ; 25 × 75 × 1.0 mm) and coverslipped with non-autofluorescent mounting medium (FluorSave reagent, Merck, #345789-20ML). Slide-mounted sections of immunolabeled hippocampi were analysed with an Olympus FluoView 1000 confocal laser microscope equipped with the following objectives: 10× (NA: 0.25), 20× (NA: 0.75), 40× (oil differential interference contrast, NA: 1.30), or 60× (oil differential interference contrast, NA: 1.35). Images were obtained with the corresponding Olympus FV10-ASW imaging software (RRID:SCR_014215) for visualization and image acquisition in a single-channel scan mode as z stacks, using 405, 473, 559, and 633 nm lasers. The resulting images (Olympus.oib format) were processed using ImageJ (RRID:SCR_003070) and projected as either maximum or average intensity (indicated in the figure legends for all images shown in this study). Super resolution images were obtained with an Elyra S.1 structural illumination microscopic (SIM) setup (Carl Zeiss) and ZEN 2.1 SP-1 image acquisition software (Carl Zeiss). Brightness and contrast were adapted, as indicated in Table 2. The γ correction was not changed in any case. Finally, the data were transferred into tif format, arranged with Adobe Illustrator software (RRID:SCR_010279), and saved as 300 dpi png and tif files.

**Table 2:** Adaption of brightness and contrast

	DAPI	Mouse-anti-BDNF-550	Guinea pig-anti-Synaptophysin-647
Hippocampus—10 ×	–	500–3304	600–4095
Hippocampus—20 ×	–	1001–4095	1301–3504

*Neuronal density and soma size determination* To evaluate the gross brain morphology of mice, every fourth vibratome section (30 μm), prepared as described above, was collected into 1× PBS, mounted on slides (Thermo Fisher Scientific; #J1800AMNZ), counterstained with cresyl violet (CertistainR, #105235, Merck) and coverslipped with GlasTM Tissue MountTM (Tissue-Tek, # 1467, Sakura). Cresyl violet stained sections were imaged using the brightfield channel of a compact fluorescence microscope (Keyence BZ-9000, BioRevo) with a 2× and 10× objective. For cell density and soma size determination, vibratome sections were stained with 4′6′-diamidino-2-phenylindole (DAPI, 1:5000), and DAPI-stained nuclei were counted manually for CA1, CA3 and DG in an area of 100,000 μm^2^ from pictures obtained by using an Olympus Fluoview confocal microscope with a 40× objective. The density of cells was calculated and the soma size (μm^2^) of those cells was measured using Fiji (RRID: SCR_002285). The dentate gyrus (DG), cornu ammonis 1 and 3 (CA1 and CA3) were identified according to Franklin and Paxinos [56].

### Immunoprecipitation and western blot

Immunoprecipitations were performed as previously described [69]. Briefly, lysates were precleared by incubation with 30 μl of protein Agarose A beads (Roche, #11719408001) on a rotator for 1 h at 4 °C. Pre-cleared lysates were then incubated with an antibody against Igf-1r (Cell Signaling Technology, #3027, RRID: AB_2122378) overnight on a rotator at 4 °C. The following day, lysates were incubated with 30 μl protein A Agarose beads (Roche, #11719408001) for 3 h at 4 °C on a rotator. Protein coupled beads were pelleted by centrifugation and washed in cold lysis buffer (150 mM NaCl, 1% Triton-X-100, 2 mM EDTA, 50 mM Tris, pH 7.4) five times and then used for Western Blot analysis. Western blots were prepared as previously described [69] with slight modifications. Briefly, hippocampi, anterior and posterior cortices were dissected, weighed, and stored at − 80 °C. Five vol/wt of lysis buffer (150 mM NaCl, 1% Triton-X-100, 2 mM EDTA, 50 mM Tris, pH 7.4) was added. To prevent proteolysis, one tablet of a protease inhibitor cocktail (Roche, #05892970001) was added to the buffer. The tissues were sonicated (Hielscher), the homogenates were then centrifuged (20,000× *g*, 20 min, 4 °C), and the supernatants collected. Samples were separated on a 8% polyacrylamide gel [66] and transferred to a PVDF membrane (Millipore). After blocking for 1 h at room temperature in 5% BSA (Fraction V, Applichem, #A13910100), membranes were incubated with primary antibodies overnight at 4 °C. The primary antibodies used were Igf-1 receptor beta (Cell Signaling Technology, Cat #3027, RRID: AB_2122378), phosho-Igf-1 receptor beta (Cell Signaling Technology, Cat #3918, RRID: AB_10548764, TrkB (Millipore, Cat # 07-225, RRID: AB_310445), pTrkB (Cell Signaling Technology, Cat # 4621, RRID: AB_916186). After three 10 min washes in 1x TBS-T (100 mM Tris–HCl, 1.5 M NaCl, 0.5% Tween-20, pH 7.5), membranes were incubated for 1 h at room temperature in corresponding secondary horseradish peroxidase-conjugated antibodies (Jackson ImmunoResearch Laboratories, Inc). Blots were developed by chemiluminescence [59, 63] (ECL or ECL prime, GE Healthcare, Lifesciences) according to the manufacturer’s instructions. Obtained blots were scanned and band intensities were quantified by densitometry analysis with Fiji (RRID: SCR_002285) [67]**.** To compare the intensities of bands from different blots, they need to be normalized to the signal from one band in each blot. Signal from the hippocampus and anterior cortex was used as a reference for pIGF-1r, Igf-1r, pTrkB, and TrkB from either 3-week, 3-month, or 18-month-old wild-type mice to compare changes at different postnatal ages as well as to determine the influence of running behavior on Igf-1r and TrkB activation.

### Statistical analysis

Data were analysed and plotted by GraphPad Prism 9.3.1 (GraphPad Software, Inc. USA, RRID: SCR_000306) and presented as mean ± SD. Statistical significance was set at *p* < 0.05. The minimum animal numbers in all behaviour experiments were calculated a priori using G*Power 3.1.9.4 software (G*Power, Heinrich Heine University Duesseldorf, Germany, RRID: SCR_013726). All statistical analyses and n of different experimental groups are reported in the respective results and figure legends. Final figures were arranged using Fiji (RRID: SCR_002285), Adobe Photoshop (RRID: SCR_014199) and Adobe Illustrator (RRID: SCR_010279).

## Results

### IGFBP5 accumulates in hippocampal pyramidal neurons and in plaques of human Alzheimer brain

Enhanced levels of Insulin-like Growth Factor Binding Protein 5 (IGFBP-5) are associated with a faster rate of cognitive decline in Alzheimer’s disease (AD) [8, 14, 45, 82]. These findings suggest that IGFBP5 may be substantially involved in the pathomechanisms at the onset of Alzheimer’s disease. To assess a possible impact of an Aß42-induced increase of IGFBP5 expression on AD pathology, we investigated the expression and distribution of IGFBP5 protein in the brains of 13 AD cases compared to 4 age-matched controls (Table 1). These cases included one patient with AD Braak II and diabetes. All cases listed in Table 1 were also tested for the presence of hippocampal TDP-43 pathology by performing immunohistochemical staining for TAR DNA-binding protein 43 (TDP-43) [44, 53]. Additional file 1: Fig. 1 shows TDP-43-positive neuronal cytoplasmic inclusions (arrows) in dentate gyrus granule cells in a case with frontal lobe dementia (FTLD, established positive internal laboratory control) and in a case with Alzheimer disease (case 15, Braak VI, CERAD C) positive for hippocampal TDP-43 pathology. Additional file 1: Fig. 1 further shows immunostaining from another Alzheimer's disease case (case 13, Braak V, CERAD C) that did not have neuronal TDP-43-positive cytoplasmic inclusions in dentate gyrus granule cells (negative for hippocampal TDP-43 pathology). Of a total of 17 cases examined for hippocampal TDP-43 pathology (4 controls and 13 AD cases), only one Alzheimer case (case 15, Braak VI, CERAD C) was found to exhibit hippocampal TDP-43 pathology. The results of all cases tested for hippocampal TDP-43 pathology are summarized in Table 1.

**Fig. 1:**
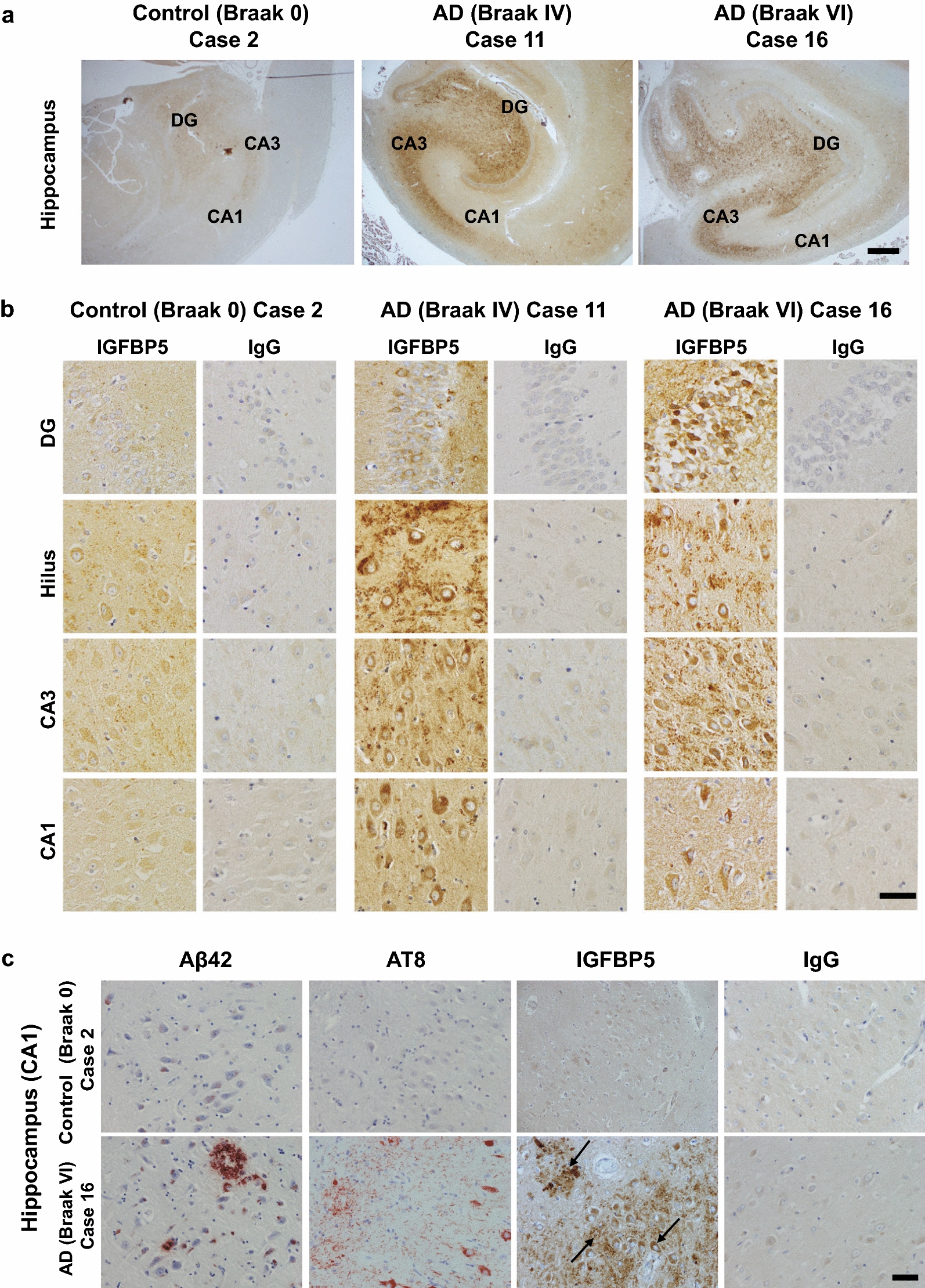
IGFBP5 accumulates in CA1 pyramidal neurons and around plaques in the hippocampus of Alzheimer cases (AD). **a** IGFBP5-IR in the hippocampus of control case 2 and AD cases 11 (Braak IV) and 16 (Braak VI) visualized by DAB immunohistochemistry. **b** IGFBP5-IR visualized by DAB immunohistochemistry in granule cells of the dentate gyrus, neuronal cells of the hilus region, CA3 and CA1 hippocampal fields of control case 2 and AD cases 11 (Braak IV) and 16 (Braak VI). **c** Representative images of Aß42, AT8, IGFBP5 and IgG control DAB immunostaining in the hippocampus and prefrontal cortex of control case 2 and AD case 16. CA, cornu ammonis; DG, dentate gyrus. Scale bars a = 200 µm; b = 50 µm; c = 50 µm

Immunohistochemical staining of hippocampal brain sections of AD cases revealed increased IGFBP5-IR in the hippocampus at an early stage of AD (Fig. 1a; Additional file 1: Fig. 2, case 5–10). Even in the end-stage AD brain (Braak VI), IGFBP5-IR remained high in the hippocampus (Fig. 1a, Additional file 1: Fig. 2, case 11–17). Strong IGFBP5-IR was observed in the dentate gyrus, the hilus region and in CA3/CA1 pyramidal neurons of the hippocampus (Fig. 1b, Additional file 1: Fig. 2). IGFBP5-IR was localized within CA1 pyramidal neurons, in the extracellular matrix and around senile plaques surrounding Aβ42 deposits (Fig. 1c, Additional file 1: Fig. 3). IGFBP5 is known to be upregulated in diabetes in many organs and tissues, including the nervous system [69]. Since comorbidity of AD and diabetes is frequently observed [17], we also studied the brain of an AD case who had diabetes (case 6, Braak II, Additional file 1: Fig. 2). Both AD cases with and without diabetes showed strong IGFBP5 expression in the hippocampus at early stages of the disease, with slightly increased IGFBP5 expression in the AD case with diabetes compared to cases without diabetes (Additional file 1: Fig. 2 cases 5–10). In contrast to the hippocampus, immunohistochemical staining of prefrontal cortical brain sections of AD cases 11 and 16 revealed no increased IGFBP5-IR compared to control case 2 (Additional file 1: Fig. 4). In the prefrontal cortex of AD case 16 almost no IGFBP5 accumulation was found around senile plaques surrounding Aβ42 deposits (Additional file 1: Fig. 4). Weak IGFBP5-IR was also detected in vascular walls of cortical blood vessels in control cases 1 and 2 and in AD cases 11 and 16, whereas no IGFBP5-IR was detected in hippocampal blood vessels in all cases listed in Table 1 (Additional file 1: Fig. 5). IGFBP5-IR further appeared in pearl-shaped structures in the border regions of blood vessels in the hippocampus and prefrontal cortex (Additional file 1: Fig. 5).

To test whether IGFBP5 labeling appears in plaque-associated dystrophic neurites in the hippocampus of AD cases, we performed double immunohistochemical staining for IGFBP5 and ubiquitin on hippocampal sections of control and AD cases, since ubiquitin is known to co-localize with neurofibrillary tangles, neuritic plaques, and neuropil threads in Alzheimer brain [60]. In control cases weak IGFBP5 immunoreactivity was visualized in CA1 pyramidal neurons, but no ubiquitin positive tangles and plaques were detected (Additional file 1: Fig. 6a–c, showing case 2 that is representative for all control cases listed in Table 1). In Braak stage I–III cases, colocalization of IGFBP5 with ubiquitin immunoreactivity was detected in typical flame-like neurofibrillary tangles localized in the CA1 region of the hippocampus (Additional file 1: Fig. 6d–i). In Braak stage IV–VI cases colocalization of IGFBP5 with ubiquitin immunoreactivity was detected in CA1 pyramidal neurons (Additional file 1: Fig. 6k, p, o) and strongly stained IGFBP5 positive CA1 pyramidal neurons were found near to ubiquitin positive plaques and tangles (Additional file 1: Fig. 6k, j, m, n). The presence of IGFBP5-IR in the hippocampus of AD brains strengthens the argument for a possible pathogenic role of IGFBP5 in AD, and the prominent expression in hippocampus could impair hippocampus-related cognitive functions.

### Voluntary wheel running increases Bdnf protein levels in the brain of wild type but not of *Bp5 tg* + mice

Igfbp5 expression is upregulated in a mouse model of AD [8]. This previous study showed that Igfbp5 is elevated in cortical pyramidal cells. In the hippocampus, Igfbp5 immunoreactivity appeared already high in the mossy fibers of wild-type control mice but also in plaque like structures in cornu ammonis 3 (CA3) and cornu ammonis 1 (CA1) of TgCRND8 mice. No Igfbp5 upregulation was observed in reactive astrocytes and microglia. Thus, elevated Igfbp5 expression in this mouse model resembles the upregulation in hippocampal pyramidal neurons and the accumulation in plaques that is a central finding in the brain sections from AD cases shown in Fig. 1, Additional file 1: Figs. 2, 3, 6j–p). Previously, we established a transgenic mouse model in which Igfbp5 is overexpressed specifically in neurons under the control of the human neurofilament-light chain (NF-L) promotor (Additional file 1: Supplementary Material) [68, 69]. Igfbp5 is a secretory protein, and it is usually released and attaches to the surrounding extracellular matrix [9, 43]. It thus can scavenge Igf-1 and prevent its association with Igf-1 receptors on the same cells that produce Igfbp5 [9, 20, 32]. In a previous study, [69], we found that Igfbp5 overexpressing mice show decreased Igf-1r activation that corresponds to neurodegeneration and myelination defects [69]. In the present study, we observed enhanced Igfbp5-IR in neuronal cell bodies of the hilus, CA3, and CA1 regions of the hippocampus and in cortical layers II/III and V of *Bp5 tg* + mice compared to wild-type mice (Additional file 1: Fig. 7a). Wet weights of total brain as well as body weights showed no significant differences between 3-, 12- and 18-month-old wild-type mice and between age-matched wild-type and *Bp5 tg* + mice (*p* > 0.05) (Additional file 1: Fig. 7b). Cresyl violet staining of coronal brain sections showed no differences in the gross brain and hippocampal morphology of wild-type and *Bp5 tg* + mice (Additional file 1: Fig. 7c). DAPI (4′,6′-diamidine-2′-phenylindoledihydro-chloride) stained brain sections were further used to determine soma size and cell density in the dentate gyrus (DG), CA3, and CA1 hippocampal regions of 3- and 13-month-old wild-type and *Bp5 tg* + mice (Additional file 1: Fig. 7 d). In all three analysed regions neither the soma size nor the cell density in 3- and 13-month-old *Bp5 tg* + mice was significantly changed compared to wild-type mice (*p* > 0.05) (Additional file 1: Fig. 7e–h). To test whether increased neuronal Igfbp5 expression had an impact on endogenous Bdnf protein levels, these were determined by ELISA in the hippocampus and anterior and posterior cortex of 3-, 12-, and 18-month-old wild-type and *Bp5 tg* + mice. Our biochemical analysis indicated that enhanced neuronal Igfbp5 expression had no effect on endogenous Bdnf protein levels in the aforementioned brain regions (*p* > 0.05) (Additional file 1: Fig. 7a, i–k). Moreover, our data show that Bdnf protein levels in the hippocampus and cortices remain constant between 3-, 12-, and 18 months (*p* > 0.05) (Additional file 1: Fig. 7i–k). In summary, increased Igfbp5 expression had no significant impact on the gross brain morphology and endogenous Bdnf protein levels.

Exercise-driven increases of hippocampal and cortical Bdnf levels are controlled by neuronal activity and peripheral factors such as Igf-1. Blocking Igf-1r signaling in the hippocampus attenuates exercise induced Bdnf expression [19, 24, 26, 30, 35, 54]. First, we provided 3-, 12- and 18-month-old wild-type mice with free access to running wheels for seven days to assess the effects of physical exercise on Bdnf protein levels (Additional file 1: Fig. 8). We found that the distance voluntarily completed during the day decreased significantly with age in 3-, 12-, and 18-month-old wild-type mice (*p* < 0.001) (Additional file 1: Fig. 8a). Next, we determined Bdnf protein levels by ELISA in exercising aging wild-type mice (Additional file 1: Fig. 8b–d). Increased Bdnf protein levels were detected in the hippocampus (*p* < 0.001) and anterior cortex (*p* < 0.01), but not in in the posterior cortex (*p* > 0.05) of 3-month-old voluntarily exercising wild-type mice (Additional file 1: Fig. 8b–d). Further we found that Bdnf protein levels neither increased in the hippocampus nor in the anterior and posterior cortex of 12- and 18-month-old exercising aged wild-type mice (*p* > 0.05) (Additional file 1: Fig. 8b–d).

**Fig. 2:**
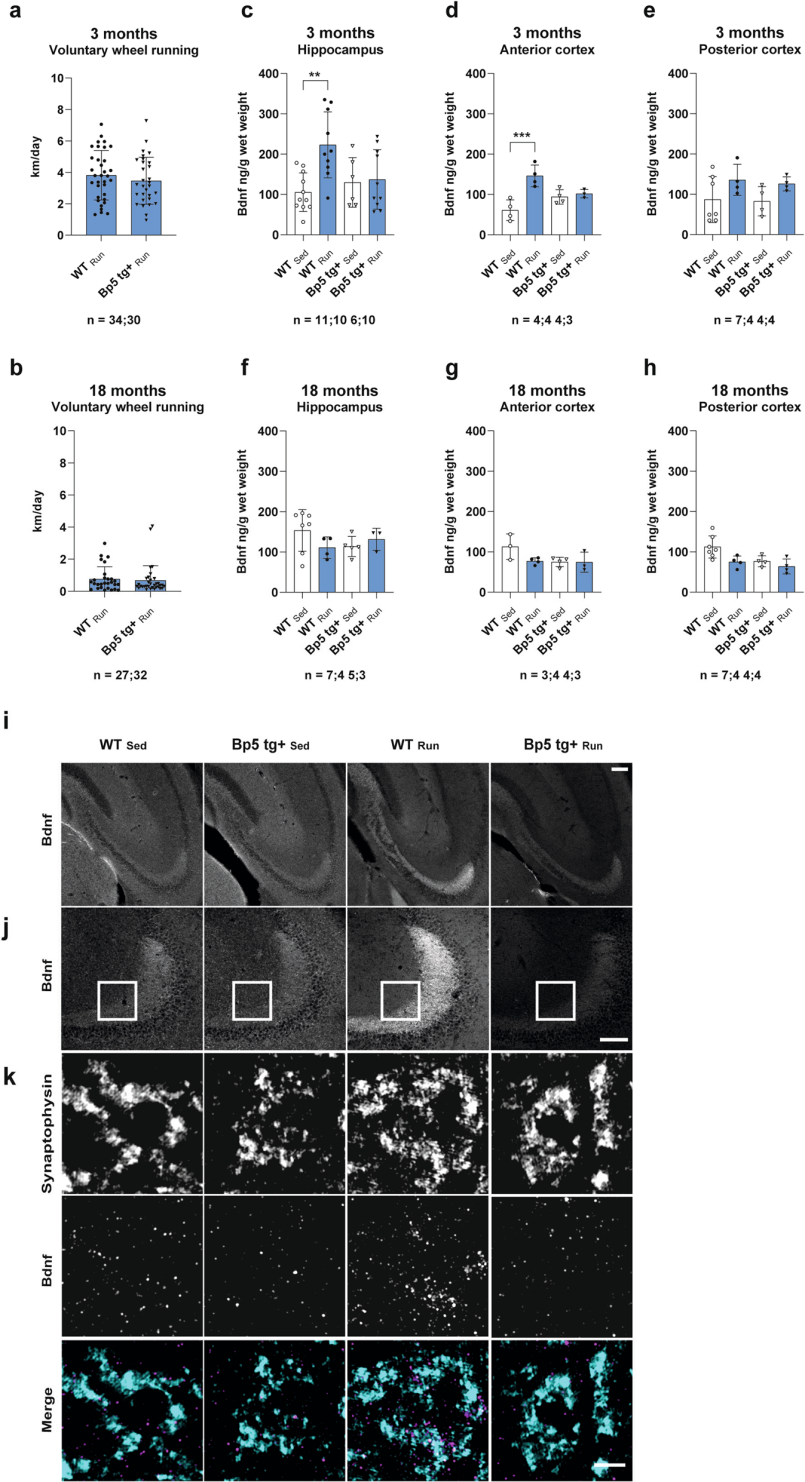
7-day voluntary wheel running increases Bdnf protein levels in the hippocampus and anterior cortex of wild-type but not of *Bp5 tg*+ mice. **a** Running distance per day of 3-month-old wild-type and *Bp5 tg*+ mice. **b** Running distance per day of 18-month-old wild-type and *Bp5 tg*+ mice. **c**, **d**, **e** Bdnf protein levels determined by ELISA in the hippocampus, anterior and posterior cortex of 3-month-old wild-type and *Bp5 tg*+ mice 7 days after voluntary wheel running. **f**, **g**, **h** Bdnf protein levels determined by ELISA in the hippocampus, anterior and posterior cortex of 18-month-old wild-type and *Bp5 tg*+ mice 7 days after voluntary wheel running. **i** Hippocampal sections of 3-month-old sedentary wild-type (WT_Sed_), running wild-type (WT_Run_), sedentary *Bp5 tg*+ (*Bp5 tg*+ _Sed_) and running *Bp5 tg*+ *(Bp5 tg*+ _Run_) mice stained for Bdnf. Intense Bdnf staining detected in the mossy fiber pathway of WT_Run_ mice compared to WT_Sed_ mice. 7-day of voluntary wheel running did not increase Bdnf intensity in the mossy fiber pathway of *Bp5 tg*+ _Run_ mice. **j** Higher magnification view of the CA3 region of WT_Sed_, WT_Run_, *Bp5 tg*+ _Sed_, *Bp5 tg*+ _Run_ sections stained for Bdnf. Note the intense staining in the stratum lucidum of CA3, containing the axon terminals of mossy fibers. **k** Super resolution microscopy images of a single CA3 neuron labeled with antibodies against Synaptophysin and Bdnf of WT_Sed_, WT_Run_, *Bp5 tg*+ _Sed_ and *Bp5 tg*+ _Run_ sections. A higher Bdnf intensity was found at the presynapse of WT_Run_ compared to WT_Sed_. Bdnf intensity of *Bp5 tg*+ _Run_ appeared comparable to that of WT_Sed_ and *Bp5 tg*+ _Sed_ mice. Data represents mean ± SD, **a**, **b** = unpaired *t-test*; **c**, **d**, **e**, **f**, **g**, **h** = One-way ANOVA, Tukey’s multiple comparison tests; **p < 0.01, ***p < 0.001; n = number of mice; Scale bars **i** = 200 µm, **j** = 50 µm, **k** = 10 µm

Next, we investigated whether enhanced expression of neuronal Igfbp5 modulates Bdnf protein levels after voluntary exercise. Therefore, 3- and 18-month-old wild-type (WT) and Igfbp5 transgenic (*Bp5 tg*+) mice were either provided with free access to running wheels for seven days or were housed in standard cages without running wheels to assess the effects of physical exercise on Bdnf protein levels (Fig. 2). The daily distance run of 3-month or 18-month-old WT and *Bp5 tg*+ mice did not differ (*p* > 0.05) (Fig. 2a, b), but decreased significantly between the ages of 3 and 18 months (*p* < 0.001). Next, we determined Bdnf protein levels by ELISA in the hippocampus and cortex of 3- and 18-month-old wild-type and *Bp5 tg*+ mice following the exercise paradigm (Fig. 2). According to ELISA quantification, Bdnf protein levels were significantly upregulated in the hippocampus after an exercise period of seven days in 3-month-old wild-type, but not in *Bp5 tg*+ mice (*p* < 0.01) (Fig. 2c). A similar effect in the exercise-induced upregulation of Bdnf was observed in the anterior cortex (*p* < 0.001) (Fig. 2d), but not in the posterior cortex (*p* > 0.05) (Fig. 2e) of 3-month-old mice. In contrast, Bdnf protein levels were not significantly upregulated by voluntary exercise in the hippocampus and anterior cortex of 18-month-old wild-type and *Bp5 tg*+ mice (*p* > 0.05) (Fig. 2f, g, h). To confirm these findings, hippocampal tissue sections were prepared from 3-month-old wild-type and *Bp5 tg*+ mice and incubated with the monoclonal Bdnf antibody Mab#9 and with polyclonal antibodies recognizing the synaptic vesicle marker synaptophysin (Fig. 2i, j, k). Bdnf immunoreactivity (Bdnf-IR) was visualized in the cell bodies and axon terminals of the mossy fiber pathway (Fig. 2i). Exercise increased Bdnf-IR clearly in the mossy fiber pathway of wild-type, but not of *Bp5 tg*+ mice (Fig. 2i). Mossy fibers project through and terminate in the stratum lucidum of CA3 and are characterized by prominent specialized endings known as mossy fiber boutons. A strong Bdnf-IR was observed within stratum lucidum (Fig. 2j). Structured illumination microscopy (SIM) revealed intense presynaptic labeling of Bdnf in mossy fiber boutons, which was confirmed by co-localization with synaptophysin. Exercise increased Bdnf-IR clearly at the mossy fiber boutons of wild-type, but not of *Bp5 tg*+ mice (Fig. 2k). The presynaptic localization of Bdnf is consistent with previous results published by Dieni and colleagues [28]. The evidence of increased Bdnf-IR at the mossy fiber boutons of wild-type but not of *Bp5 tg*+ mice further highlight the results of Bdnf ELISA readings in the hippocampus of 3-month-old exercising wild-type and *Bp5 tg*+ mice (Fig. 2c).

## Impaired cognitive function in young *Bp5*+ exercising mice

Physical exercise enhances spatial learning and memory formation in rodents [39, 73] and this effect is due to the induction of Bdnf expression in the hippocampus [24, 52]. In the present study, we investigated the effects of enhanced neuronal Igfbp5 expression on spatial memory in aging voluntary exercising mice. Initially, 3- and 18-month-old wild-type and *Bp5 tg*+ mice were tested in the open-field arena after 7 days of voluntary wheel running, to examine general locomotor activity and anxiety-related behaviour (Additional file 1: Fig. 9). Neither sedentary nor running 3-month-old *Bp5 tg*+ mice showed signs of hyperactivity and anxiety-related behaviour compared to age matched wild-type mice (*p* > 0.05) (Additional file 1: Fig. 9a–f). The basal activity of 18-month-old running wild-type mice was significantly decreased compared to sedentary control wild-type mice (*p* < 0.05), whereas no difference was found between 18-month-old sedentary and running *Bp5 tg*+ mice (*p* > 0.05) (Additional file 1: Fig. 9 h, i). Neither sedentary nor running 18-month-old *Bp5 tg*+ mice showed signs of anxious behaviour compared to age matched wild-type mice (*p* > 0.05) (Additional file 1: Fig. 9j–l). These results suggest that increased neuronal Igfbp5 expression and voluntary exercise did not significantly affect general motor activity, exploratory- or anxiety-like behaviour in 3- and 18-month-old mice, and thus there should be no effects of increased anxiety-like behaviour on specific memory tests.

**Fig. 3:**
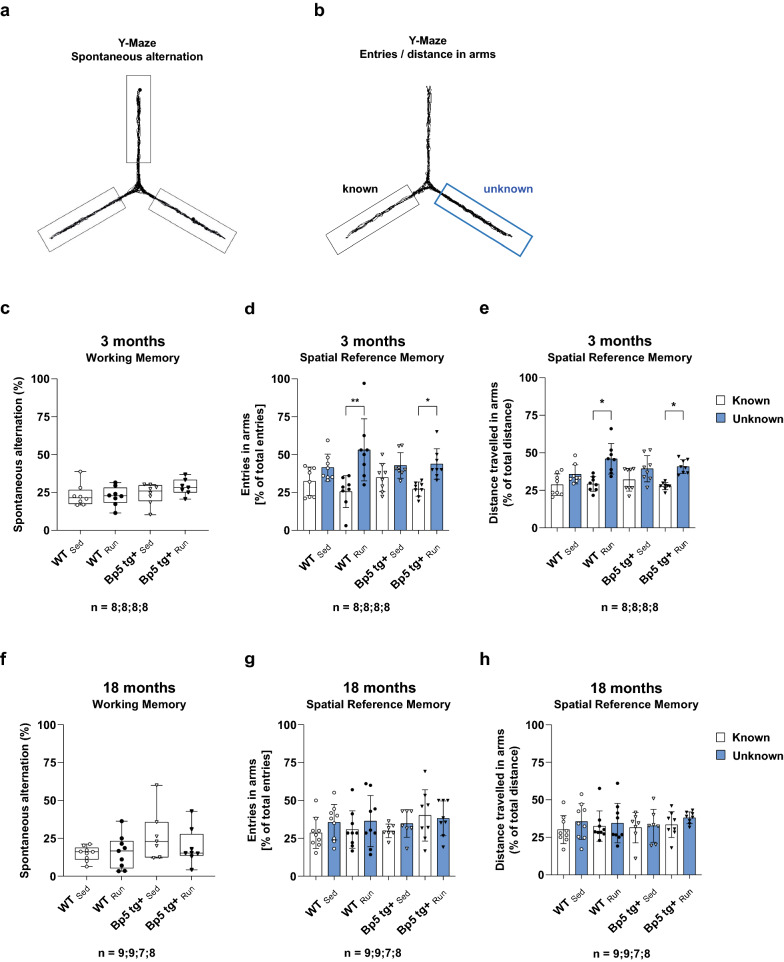
Physical exercise improves spatial reference memory in the Y-maze test of young mice. **a** Diagram for spontaneous alternations in the Y-Maze test. **b** Diagram of entries and distances to the known and unknown arm in the Y-Maze. **c** Quantification of spontaneous alternation between 3-month-old WT_Sed_, WT_Run_, *Bp5 tg* + _Sed_ and *Bp5 tg* + _Run_ mice. **d**, **e** Quantification of spatial reference memory in 3-month-old WT_Sed_, WT_Run_, *Bp5 tg* + _Sed_ and *Bp5 tg* + _Run_ mice by analysing the number of arm entries (**d**) and distance travelled (**e**) into known versus unknown arm after entering the maze. **f** Quantification of spontaneous alternation between 18-month-old WT_Sed_, WT_Run_, *Bp5 tg* + _Sed_ and *Bp5 tg* + _Run_ mice. **g**, **h** Quantification of spatial reference memory in 18-month-old WT_Sed_, WT_Run_, *Bp5 tg* + _Sed_ and *Bp5 tg* + _Run_ mice by analysing the number of arm entries (**g**) and distance travelled (**h**) into known versus unknown arm after entering the maze. Data represent mean ± SD, Kruskal–Wallis test, Dunn's post hoc test; *p < 0.05, **p < 0.01, ***p < 0.001; n = number of mice

We then tested short-term working memory in 3- and 18-month-old wild-type and *Bp5 tg*+ mice after 7 days of voluntary wheel running by spontaneous alternations in the Y-maze [47] (Fig. 3). Wild-type and *Bp5 tg*+ mice showed a similar percentage of spontaneous alternations. Voluntary wheel running and age had no effect on spontaneous alternations (*p* > 0.05) (Fig. 3c, f). When mice were given access to a new arm that was blocked during the training phase, 3-month-old exercising wild-type and *Bp5 tg*+ mice entered the new previously unvisited arm of the maze more frequently than sedentary wild-type and *Bp5 tg*+ mice (wild-type: *p* < 0.05, *Bp5 tg*+: *p* < 0.01) (Fig. 3d) Furthermore, 3-month-old exercising wild-type and *Bp5 tg* + mice travelled a longer distance in the new previously unvisited arm of the maze than did sedentary wild-type and *Bp5 tg*+ mice (*p* < 0.05) (Fig. 3e). When 18-month-old exercising wild-type and *Bp5 tg*+ mice were given access to the new arm, they did not enter the new previously unvisited arm of the maze more frequently than sedentary wild-type and *Bp5 tg*+ mice (*p* > 0.05) (Fig. 3g) and did not travel a longer distance there (*p* > 0.05) (Fig. 3h). Taken together these data indicate that 7 days of voluntary wheel running improved short-term working memory in 3-month-old, but not in 18-month-old wild-type and *Bp5 tg*+ mice.

**Fig.4:**
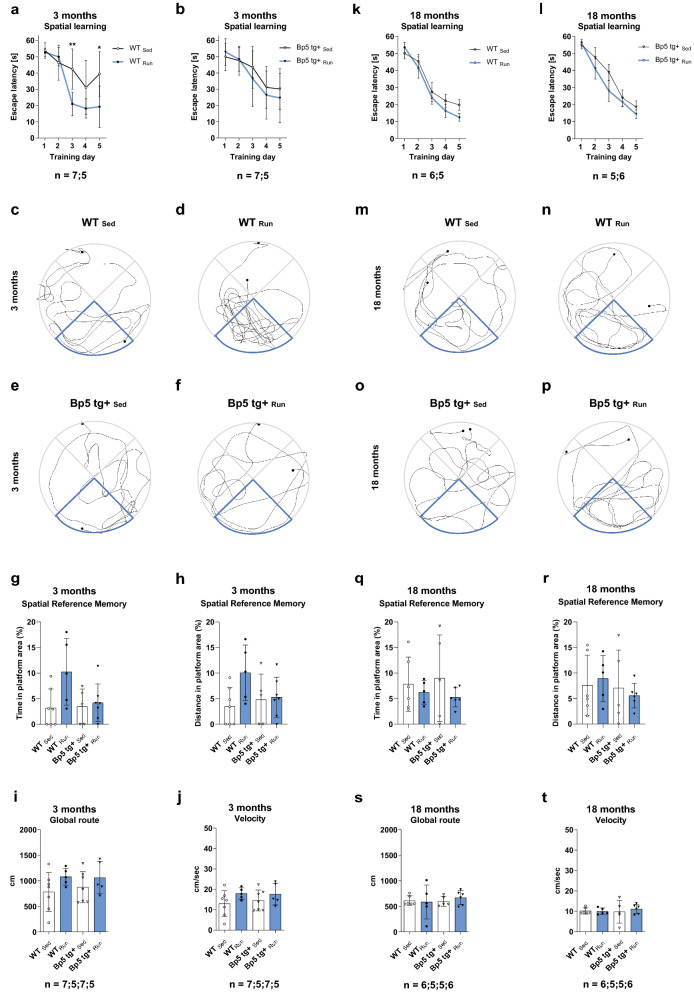
Impaired cognitive function in young exercising *Bp5 tg* + mice. **a** Escape latency of 3-month-old WT_Sed_ and WT_Run_ mice. **b** Escape latency of 3-month-old *Bp5 tg* + _Sed_ and *Bp5 tg* + _Run_ mice. **c**, **d**, **e**, **f** Representative images of the path travelled during the probe trial, illustrating the preference of 3-month-old WT_Run_ mice for the platform area compared to 3-month-old WT_Sed_, *Bp5 tg* + _Sed_ and *Bp5 tg* + _Run_ mice. **g** Time in the platform area of 3-month-old WT_Sed_, WT_Run_, *Bp5 tg* + _Sed_ and *Bp5 tg* + _Run_ mice. **h** Travelled distance in the platform area of 3-month-old WT_Sed_, WT_Run_, *Bp5 tg* + _Sed_ and *Bp5 tg* + _Run_ mice. **i** Global route of 3-month-old WT_Sed_, WT_Run_, *Bp5 tg* + _Sed_ and *Bp5 tg* + _Run_ mice. **j** Velocity of 3-month-old WT_Sed_, WT_Run_, *Bp5 tg* + _Sed_ and *Bp5 tg* + _Run_ mice. **k** Escape latency of 18-month-old WT_Sed_ and WT_Run_ mice. **l** Escape latency of 18-month-old *Bp5 tg* + _Sed_ and *Bp5 tg* + _Run_ mice. **m**, **n**, **o**, **p** Representative images of the path travelled during the probe trial showing no preference of 18-month-old WT_Run_ mice for the platform area compared to 18-month-old WT_Sed_, *Bp5 tg* + _Sed_ and *Bp5 tg* + _Run_ mice. **q** Time in the platform area of 18-month-old WT_Sed_, WT_Run_, *Bp5 tg* + _Sed_ and *Bp5 tg* + _Run_ mice. **r** Travelled distance in the platform area of 18-month-old WT_Sed_, WT_Run_, *Bp5 tg* + _Sed_ and *Bp5 tg* + _Run_ mice. **s** Global route of 18-month-old WT_Sed_, WT_Run_, *Bp5 tg* + _Sed_ and *Bp5 tg* + _Run_ mice. **t** Velocity of 18-month-old WT_Sed_, WT_Run_, *Bp5 tg* + _Sed_ and *Bp5 tg* + _Run_ mice. Data represents mean ± SD, **a**, **b**, **k**, **l** = Two-way ANOVA, Bonferroni's multiple comparison tests; **g**, **h**, **i**, **j**, **q**, **r**, **s**, **t** = One-way ANOVA, Tukey’s multiple comparison tests; *p < 0.05, **p < 0.01, ***p < 0.001; n = number of mice. Blue highlighted area = target quadrant

Complementing the Y-Maze, we performed next a four-trial-per-day, 5-day Morris water maze (MWM) paradigm [76] with 3- and 18-month-old wild-type and *Bp5 tg*+ mice after 7 days of voluntary wheel running (Fig. 4). Results of 3-month-old mice showed that the escape latencies were similar in all four groups at day one of the MWM training (*p* > 0.05) (Fig. 4a, b). Consistent with previous findings for the effect of exercise on promoting learning acquisition [73], we found that 7-days of voluntary wheel running decreased the latency to locate the hidden platform in running wild-type mice as compared with sedentary controls (Fig. 4a). The effect of exercise manifested as a decrease in latency to find the hidden platform on days 3 and 5 of the MWM training as compared with sedentary control mice (day 3: *p* < 0.01; day 5: *p* < 0.05) (Fig. 4a). However, 3-month-old sedentary wild-type mice showed impaired learning performance on day 5 compared to day 4 (Fig. 4a) in comparison to the group of running wild-type mice under the same condition. Performance during this training phase can vary when mice are exposed to distracting stimuli on one of these days, which then affects both groups in the same experiment. Enhanced levels of neuronal Igfbp5 in exercising mice failed to significantly alter the exercise-induced effect on learning acquisition on days 3 and 5 of the MWM training. Exercise had no effect on the latency to find the hidden platform on days 3 and 5 of MWM training in 3-month-old *Bp5 tg*+ mice compared to sedentary controls (*p* > 0.05) (Fig. 4b). Results of 18-month-old mice showed that the escape latencies were similar between all four groups at day 1 of MWM training and progressively decreased over 5 days of acquisition training in sedentary and exercising wild-type and *Bp5 tg*+ mice. Exercise had no effect on the latency to find the hidden platform over the 5 days of acquisition training (*p* > 0.05) (Fig. 4k, l). To evaluate memory retention, we performed a probe trial 24 h after the last MWM training day (day 6). Mice were allowed to swim for 60 s in the pool in which they received their training, but with the escape platform removed. The percentage of time and the distance spent in the platform area, which was a small part of the target quadrant, was calculated for each animal (see Additional file 1: Supplementary Material for details). We found that 3-month-old exercising wild-type mice showed a clear preference for the platform area over sedentary wild-type controls, as they spent a greater percentage of time and distance, though not significantly in the platform area than sedentary wild-type controls (*p* > 0.05) (Fig. 4c, d, g, h, Additional file 1: Fig. 10). This exercise-induced preference for the platform area was not observed in exercising 3-month-old *Bp5 tg*+ mice, such that there was no difference between the percentage of time and distance spent in the platform area by exercising *Bp5 tg*+ and sedentary control *Bp5 tg*+ mice (*p* > 0.05) (Fig. 4e, f, g, h, Additional file 1: Fig. 10). Results of 18-month-old mice showed that the exercise-induced preference for the platform area, as observed in exercising 3-month-old wild-type mice, was not detected in exercising 18-month-old wild-type mice (*p* > 0.05) (Fig. 4m, n, q, r, Additional file 1: Fig. 11). To control for differences in MWM performance, we recorded each animal’s travelled distance and swimming speed in the 3- and 18-month-old mouse cohorts used in this study. We found no difference in distances and swimming speeds between all four respective groups, ruling out motor deficits in this task (*p* > 0.05) (Fig. 4i, j, s, t) [69]. These data indicate that physical exercise enhances spatial learning, memory formation and memory recall in 3-month-old, but not in 18-month-old wild-type mice. Enhanced levels of neuronal Igfbp5 prevented the exercise-induced effect on spatial memory formation during the MWM training and the benefits of exercise on memory recall in 3-month-old mice. Enhanced levels of neuronal Igfbp5 had no influence on learning acquisition, memory formation and memory recall in 18-month-old mice.

### Reduced Igf-1r activation but consistent TrkB activation in the hippocampus of 3-month-old exercising *Bp5 tg*+ mice

**Fig. 5:**
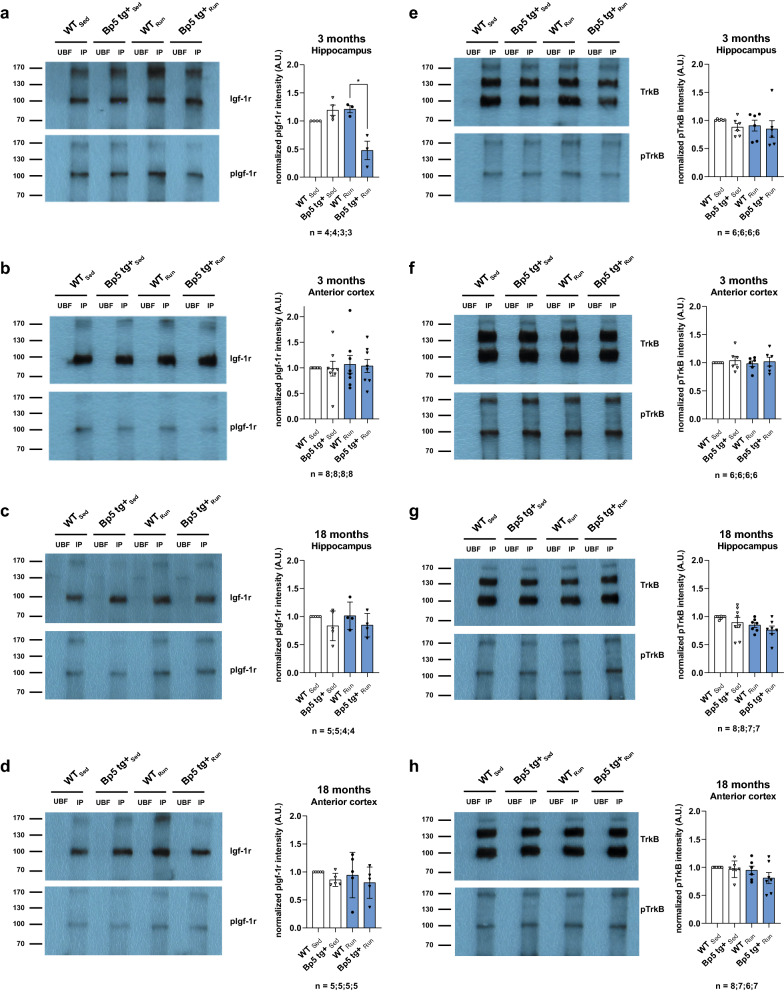
Reduced Igf-1r activation but invariant TrkB activation in the hippocampus of exercising 3-month-old *Bp5 tg* + mice. **a**–**d** Igf-1r and **e**–**h** TrkB immunoprecipitation from hippocampal and cortical lysates of 3- and 18-month-old wild-type and *Bp5 tg* + mice either sedentary or voluntarily running for 7 days. Data represent mean ± SD, ANOVA, Kruskal–Wallis test, Dunn’s post hoc test, *p < 0.05; n = number of mice. Values were normalized to 3-month-old WT_Sed_ hippocampus (**a**, **e**) or anterior cortex (**b**, **f**) or to 18-month-old WT_Sed_ hippocampus (**c**, **g**) or anterior cortex (**d**, **h**), UBF unbound fraction, IP immunoprecipitation

Blocking of hippocampal Igf-1 receptors during voluntary exercising has been reported to significantly reduce the increase of Bdnf transcription and translation, and downstream TrkB signaling, including phosphorylation of CaMKII and MAPK-II, in parallel with attenuated upregulation of proteins that regulate synaptic function, such as synapsin 1 [26, 30]. To determine the influence of enhanced levels of neuronal Igfbp5 on Igf-1r activation at 7-day after voluntary exercising, we immunoprecipitated the Igf-1r from hippocampal and cortical lysates of 3- and 18-month-old *Bp5 tg*+ mice (Fig. 5a–d). Analyses of hippocampal and cortical lysates by Western blot with a specific pIgf-1r antibody revealed a significant 60% reduction in the phosphorylation of the Igf-1r in the hippocampus (*p* < 0.05), but not in the anterior cortex (*p* > 0.05) of exercising 3-month-old *Bp5 tg*+ mice compared to exercising wild-type mice (Fig. 5a, b). Physical activity did not significantly increase pIgf-1r levels in the hippocampus and anterior cortex of 3-month-old wild-type mice. Enhanced levels of Igfbp5 had no measurable effect on Igf-1r phosphorylation in the hippocampus and anterior cortex of 3-month-old sedentary *Bp5 tg*+ mice (*p* > 0.05) (Fig. 5a, b). Analyses of hippocampal and cortical lysates of 18-month-old mice indicated that neither physical activity nor neuronal Igfbp5 had an influence on Igf-1r activation (*p* > 0.05) (Fig. 5c, d). Furthermore, we immunoprecipitated TrkB from hippocampal and cortical lysates of 3- and 18-month-old *Bp5 tg*+ mice to determine the influence of enhanced levels of Igfbp5 on TrkB activation at 7-day after voluntary wheel running (Fig. 5e–h). Analyses of hippocampal and cortical lysates by Western blot with a specific pTrkB antibody revealed no alteration in the phosphorylation of TrkB in the hippocampus and anterior cortex of 3- and 18-month-old exercising *Bp5 tg*+ mice compared to exercising wild-type controls (*p* > 0.05). Neither physical activity nor neuronal Igfbp5 influenced the level of TrkB phosphorylation at 7 days after voluntary exercise initiation. In summary, the immunoprecipitation data show that increased expression of neuronal Igfbp5 decreased Igf-1r activation in the hippocampus in 3-month-old but not in 18-month-old exercising *Bp5 tg*+ mice and those 7 days of voluntary wheel running did not increase Igf-1r activity in the hippocampus of 3-month-old wild-type mice.

### Bdnf levels do not correlate with TrkB and Igf-1r activation

**Fig. 6:**
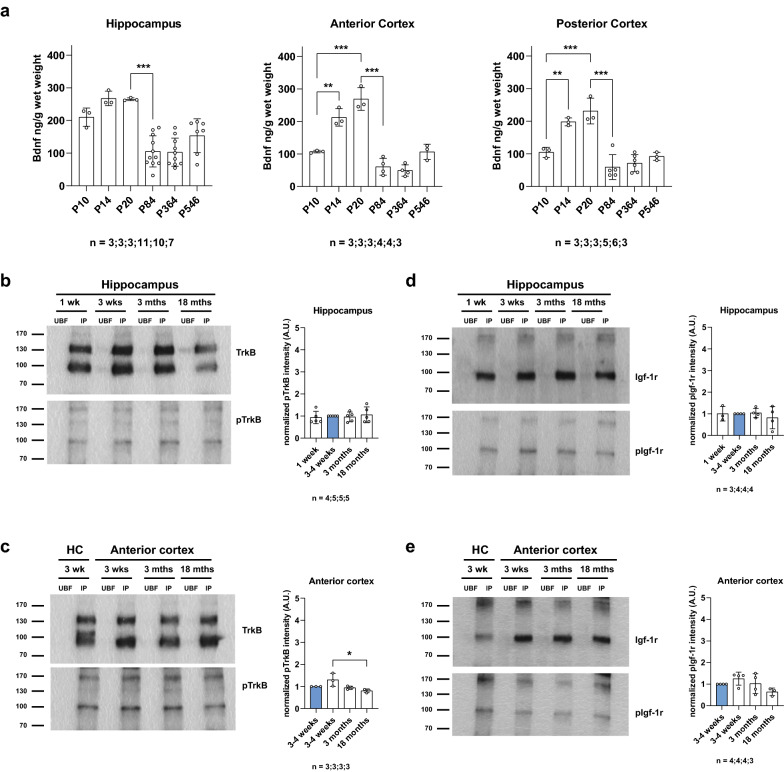
Bdnf levels do not correlate with TrkB and Igf-1r activation. **a** Bdnf protein levels determined by ELISA in the developing and aging hippocampus, anterior and posterior cortex of wild-type mice. Data represent mean ± SD, One-way ANOVA, Tukey’s post hoc test; *p < 0.05, **p < 0.01, ***p < 0.001. **b**–**e** TrkB and Igf-1r immunoprecipitation from hippocampal and cortical lysates of 1 week, 3–4 weeks, 3- and 18-month-old wild-type mice. Data represent mean ± SD, ANOVA, Kruskal–Wallis test, Dunn’s post hoc test; *p < 0.05; n = number of mice. Values were normalized to 3-week-old wild-type hippocampus (blue column), UBF unbound fraction, IP immunoprecipitation

Endogenous Bdnf protein levels increase during brain development and are highest in the brain of 3-week-old wild-type mice [46]. Bdnf protein levels have been reported to remain high but decrease in the CNS of adult mice compared to 3-week-old mice [62]. Based on these data, we asked whether there is a correlation between Bdnf protein levels and TrkB receptor activation in the hippocampus and anterior cortex of 3-week-old mice, and whether reduced Bdnf protein levels in the adult hippocampus and anterior cortex affect TrkB receptor activation. First, we determined Bdnf protein levels in the hippocampus and cortex of developing and ageing wild-type mice by ELISA (Fig. 6a). In line with previous reports, Bdnf protein levels were significantly higher in the hippocampus and cortex of 3-week-old wild-type compared to 3-month-old mice (*p* < 0.001). To determine whether Bdnf protein levels in the hippocampus and cortex of 3-week-old wild-type mice were associated with a maximum of TrkB receptor activation, we immunoprecipitated TrkB from hippocampal and cortical lysates of 1-week, 3–4 weeks, 3- and 18-month-old wild-type mice (Fig. 6b, c). Analyses of hippocampal lysates by Western blot with a specific pTrkB antibody revealed no alteration in the phosphorylation of TrkB in the hippocampus of 1 week-, 3- and 18-month-old wild-type mice compared to 3–4 weeks old wild-type mice (*p* > 0.05). Analyses of anterior cortex lysates revealed a significant 38% reduction in the phosphorylation of TrkB in the anterior cortex of 18-month-old wild-type mice compared to 3–4 weeks old wild-type mice (*p* < 0.05). Immunoprecipitation of the Igf-1r from hippocampal and cortical lysates of 1 week, 3–4 weeks, 3- and 18-month-old wild-type mice revealed no alteration in the phosphorylation of the Igf-1r at any of the measured time points (*p* > 0.05) (Fig. 6d, e). These data indicate that the levels of TrkB phosphorylation and possibly also of Igf-1r do not correlate with the levels of Bdnf and Igf-1 that are expressed and available to these receptors. This could be due to regulatory effects such as modulation of number of receptors at the neuronal cell surface [3] or downstream effects such as the activation of receptor specific phosphatases that dephosphorylate these receptors after activation. Since the activation of TrkB and Igf-1r was tested at 7 days after initiation of voluntary exercise and not within a short interval after training onset, such counter-regulatory effects could come into play. To test this possibility, we analyzed hippocampal and cortical lysates from 3-week-old *Bdnf* heterozygous knockout mice (*Bdnf*^+/−^) in which Bdnf levels are reduced by roughly 50%. Brain lysates from these mice did not show any changes in TrkB and Igf-1r activation compared to control lysates of 3-week-old wild-type mice (p > 0.05) (Additional file 1: Fig. 12a, b), thus providing further evidence that the levels of steady state TrkB activation do not correlate with Bdnf levels in brain.

## Discussion

Our study reveals that IGFBP5 accumulates in pyramidal neurons and in plaques of Alzheimer patients localized in the hippocampus. Neuronal overexpression of Igfbp5 prevents the training-induced increase of hippocampal and cortical Bdnf protein levels and diminishes the effects of exercise on memory retention, but not on learning acquisition. Igfbp5 prevents hippocampal Igf-1r activation in exercising mice but has no influence on total levels of TrkB activation. This effect was most prominent in 3-month-old mice when age-dependent cognitive decline is not expected. At this early age, the effect of Igfbp5 overexpression on antagonizing the effects of physical exercise on Bdnf upregulation also appears most pronounced. This correlates with reduced performance in spatial memory recall. Similarly, the increase of IGFBP5 immunoreactivity in Alzheimer brain also appears more pronounced at early stages of disease in comparison to later Braak stages. Thus, elevated levels of Igfbp5 limit the exercise induced Bdnf upregulation and thereby Bdnf mediated plasticity changes in the hippocampus and possibly also in other areas of the brain that are involved in spatial memory storage and recall. Hence, elevated IGFBP5 expression could be responsible for some of the early cognitive deficits that occur during the course of Alzheimer’s disease. Future studies should clarify whether the observed upregulation of IGFBP5 is specific for AD or whether an increase in IGFBP5 expression is generally expected in neurodegenerative diseases or brain injury. Based on previous studies, increased IGFBP5 expression in the prefrontal cortex of PD or depression has been suggested. However, to date, there are no systematic histological studies to support this finding [18, 82].

### Mechanisms for upregulation of IGFBP5 in the AD brain

Why do neurons upregulate IGFBP5 expression to limit their capacity for plasticity of synaptic circuits and synapses such as the mossy fiber terminals? One could speculate that neurons protect themselves against excessive metabolic demands that are associated with high activity and plasticity induced structural and functional changes at synapses. This could explain the high upregulation of IGFBP5 protein levels at early stages of Alzheimer Disease when synaptic plasticity still could occur [16]. At early stages of Alzheimer’s Disease, enhanced neuronal activity is frequently observed and appears as a common pathomechanism [16, 78]. Thus, inhibition of IGF-1 and suppression of mechanisms that upregulate BDNF expression could represent a compensatory process. Enhanced expression of Igfbp5 limits the upregulation of Bdnf in the hippocampus and anterior cortex in transgenic mice, and thus the capacity for synaptic plasticity.

Intra-neuronal accumulation of Aβ is among the earliest known events in AD pathogenesis, preceding the appearance of both neurofibrillary tangles and amyloid plaques [40, 71, 79]. Work by Barucker and colleagues proposes a pathophysiological cascade that begins with Aβ42-low-n oligomerization in the nucleus and induces Igfbp5 expression in brains of AD mice at the pre-plaque stage [7, 8]. The significance of this finding is further supported by the observation of increased IGFBP5 levels in the CSF from AD patients (> 60 years of age) [8, 65]. Thus, our finding of increased IGFBP5 expression in pyramidal neurons in the hippocampus of AD brains is in line with previous findings that IGFBP5 and altered IGF signaling in general are associated with AD [21, 37, 72]. Reduced bioavailability and activity of IGF-1 due to increased neuronal IGFBP5 expression in the hippocampus may also reduce AD-associated hippocampal hyperactivity at early stages of AD progression due to reduced IGF-1R activation [4, 16, 78]. This could counteract neuronal hyperactivity, but on the other side also limit the capacity for synaptic plasticity. It is possible that reduced IGF-1R activation or inhibition of elevated IGF-1R activation limits hippocampal hyperactivity in individuals who are at risk for AD and in AD patients, but this apparently comes at the expense of reduced capacity for synaptic plasticity which goes along with cognitive decline, in particular for functions such as spatial memory retention. A recent study in the field of cancer biology performed with mouse embryonic fibroblasts identified IGFBP5 as a secreted, mTORC1 downstream effector protein that inhibits the function of IGF-1. Once secreted, IGFBP5 cooperates with intracellular branches of the feedback mechanisms to block the activation of IGF-1 signaling [29]. However, this mTOR dependent IGF-1/IGFBP5 signaling pathway has not yet been studied in the hippocampus in the context of Alzheimer’s disease and needs further investigation [41, 42, 48, 58].

### Igfbp5 modulates the effects of exercise on Bdnf upregulation and memory recall

It is not clear whether physical exercise enhances neural activity of hippocampal granule cells, which then upregulates Bdnf expression, or whether the upregulation of Bdnf expression is mediated by other means, resulting then in enhanced neuronal activity and in particular in a condition that allows enhanced synaptic plasticity [75]. The very low expression of Igf-1 mRNA and the broad expression of Igf-1 receptors in the adult mammalian brain suggests that peripherally produced Igf-1 may play a role in the induction of hippocampal and cortical Bdnf expression [6, 13, 35]. This is supported by the identification of a mechanism for the activity-dependent entry of Igf-1 from the serum into active brain regions through cerebral vascular endothelial cells [35, 54]. In addition, intracarotid injection of Igf-1 has been shown to increase Bdnf expression in the hippocampus [19]. Ding and colleagues provided evidence that blocking Igf-1r signaling reverses the exercise-induced increase of Bdnf protein levels and abolishes the exercise-enhancement in memory recall but fails to influence learning acquisition [30]. The molecular analysis of this study revealed that exercise increases endogenous Igf-1 and Bdnf levels, as well as proteins downstream of Bdnf activation that are important for synaptic function. Blocking Igf-1r abolishes these exercise-induced increases [30]. These results illustrate a possible mechanism by which Igf-1 interfaces with the Bdnf system to mediate exercise-induced synaptic and cognitive plasticity [30]. Here, we show that enhanced Igfbp5 expression in pyramidal neurons of the hippocampus and cortex prevents the increase of Bdnf protein levels in 3-month-old exercising mice and the training-induced improvement of memory performance in the Morris water maze. In 18-month-old wild-type mice that were exercising voluntarily, we did not detect an increase of Bdnf protein levels in the hippocampus and anterior cortex or a training-induced improvement of memory performance in the Morris water maze. This could be because the intensity of voluntary exercise is low in 18-month-old mice, as measured by the distance that mice ran per day in the running wheel. However, similar effects were also observed in 12-month-old mice that still ran more than 3 km a day. At 12 months, upregulated Bdnf expression after exercise was only marginal, indicating that the lower exercise performance in 18-month-old mice is not the sole reason for a downscaled response in hippocampal Bdnf upregulation, and that other age-related mechanisms such as reduced activation of the Igf-Igf-1r system could come into play. Based on the study by Yang and colleagues [81], it can be hypothesized that less or no circulating Igf-1 is transported to the CNS parenchyma in 18-month-old voluntarily exercising mice due to an age-related change in physiological blood–brain transport [35, 54]. This would result in no or less acute activation of the Igf-1 receptor and downstream signaling cascades required to induce Bdnf expression [30]. However, this point needs further investigation in future studies. In summary, our data indicate that upregulated IGFBP5 expression that occurs in Alzheimer brain has major impact on the IGF-1/IGF-1R system in modulating BDNF expression and thus allowing synaptic plasticity for maintenance of cognitive processes such as memory recall. Elevated IGFBP5 protein levels have been detected in the CSF of AD patients [8, 65], but it remains to be established whether IGFBP5 levels in serum or CSF can predict adverse cognitive outcomes, including dementia risk, over time. Given the growing interest in dementia therapeutics targeting impaired insulin metabolism and IGF signaling [11, 27], IGFBP5 may be a useful circulating biomarker to predict dementia risk for future clinical trials. Therefore, future clinical trials should examine the association between IGFBP5 serum or CSF levels, structural brain magnetic resonance imaging, cognitive performance, and the incidence of dementia and AD in a large, prospective cohort of cognitively healthy adults.

## Supplementary Information

**Additional file 1.** Online Resource Supplementary Material and Figures.
