# Supplementary material for: Insulin-like growth factor 5 associates with human Aß plaques and promotes cognitive impairment
Source: Acta Neuropathol Commun. 2022 May 5;10:68. doi: 10.1186/s40478-022-01352-5 (PMC9074221; doi:10.1186/s40478-022-01352-5)
Supplement: Supplementary file 1 — Additional file 1. Online Resource Supplementary Material and Figures. [file 40478_2022_1352_MOESM1_ESM.pdf]

Online Resource Supplementary Material and Figures

Insulin-like growth factor 5 associates with human Aß plaques and promotes cognitive impairment

Running title: IGFBP5 and Alzheimer

Stefanie Rauskolb[1], Thomas Andreska[1], Sophie Fries[1], Cora Ruedt von Collenberg[1], Robert Blum[1,3], Camelia - Maria Monoranu[2], Carmen Villmann[1] and Michael Sendtner[1]

1. Institute of Clinical Neurobiology, University of Würzburg, Versbacher. Str. 5, 97078 Würzburg, Germany
2. Department of Neuropathology, Institute of Pathology, University of Würzburg, Josef-Schneider-Str. 2, 97080 Würzburg, Germany
3. Department of Neurology, University Hospital Würzburg, Josef-Schneider-Str. 11, 97080 Würzburg

Corresponding author:

Michael Sendtner

Institute of Clinical Neurobiology, University of Würzburg,

Versbacherstr. 5, 97078 Würzburg, Germany

Phone: 0049-931-201-44000

Fax: 0049-931-201-44009

mail to: Sendtner_M@ukw.de

Online Resource Supplementary Material

Generation of *Igfbp5* transgenic mice

Construct for IGFBP5 overexpression under the control of a neurofilament light chain promotor

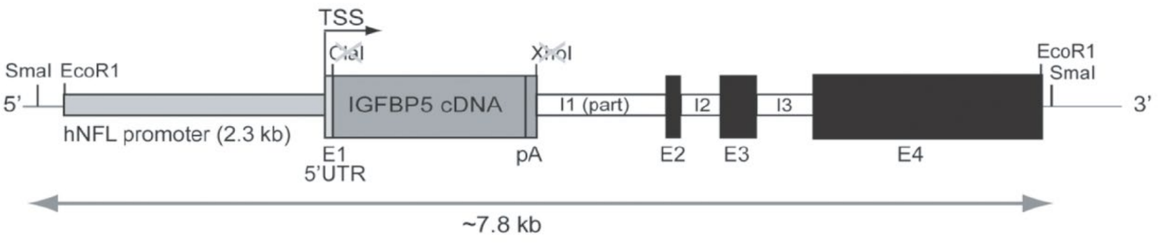

Isolation of *Igfbp5* cDNA clones

A λ-ZAPII mouse brain cDNA library was screened using an 873bp PCR-generated cDNA probe (forward primer IGFBP5-1 5'-GCC CCG AGG TAA AGC CAG ACT-3'; reverse primer IGFBP5-3 5'-GGA TAG GGG GAG GAA GGG AGG-3') representing the entire coding sequence and 48bp of 5'untranslated region. Clones with the full-length cDNA were plaque purified and high titer stocks prepared. Excision of the integrated pBS II SK vectors containing cDNA inserts in the polylinker EcoRI site was carried out using the EXASSIST/SOLR system (Stratagene, La Jolla, Ca) according to the manufacturer's instructions and clone identity was confirmed by DNA sequencing.

Construction of a NF-L-*Igfbp5* DNA targeting vector

A 1.8kb *Eco*RI fragment containing the *Igfbp5* cDNA (excised from pBSII SK using the polylinker *Eco* RV site and an NseI site in the 3´ UTR) and the polyA region from pMC-Cre (S1) were isolated and blunt-end ligated into pKS.NFL (gift from J. P. Julien) between blunted XhoI/ClaI sites. The 8kb fragment including the human *NF-L* promoter, mouse *Igfbp5* cDNA, the polyA signal from pMC-Cre and exons 2-4 of the

NF-L gene (downstream region) was excised from NFL-BP5 using *Sma*I. The fragment was gel purified twice and purified with Nucleotrap resin (Macherey and Nagel, Düren, Germany), extracted with butanol, then desalted and further purified over a Schleicher and Schuell (Dassel, Germany) Elutip. Eluted DNA was extracted with phenol/chloroform and chloroform and then precipitated with ethanol. DNA for microinjection was resuspended in sterile-filtered injection buffer (5mM Tris-HCl, pH7.4, 0.1mM EDTA) at approximately 500 copies/pl. DNA and microinjected into fertilized mouse oocytes.

Identification and characterization of *Igfbp5* transgenic mouse lines

Nine founders were identified by PCR and Southern blot analysis of tail DNA. The PCR reaction used for genotyping was carried out with forward primer NFL-SEQ 5´-TCG CAG GCT GCG TCA GGA G-3´ and reverse primer BP5PCR 5´-CTT GCA GGT AGA GCA GGT GCT CTC-3´, 45 cycles of 45" at 94°C, 45" at 53°C, 30" at 72°C. For Southern blots, tail DNA was digested with *Xho*I, and probed with the radiolabeled *Igfbp5* PCR product described above.

NFL-Cre transgenic mice

The NFL-Cre transgenic mice have been described previously (Schweizer U et al., J Cell Biol. 2002 Jan 21;156(2):287-97). Highest expression levels are found in pyramidal neurons and other large neurons such as motoneurons. A detailed analysis of the expression pattern of Cre under the NFL promoter is included in the PhD thesis of Ulrich Schweizer (https://opus.bibliothek.uni-wuerzburg.de/opus4-wuerzburg/frontdoor/deliver/index/ docId/311/file/Diss.Schweizer.PDF ttf.doc (uni-wuerzburg.de), and table 3.2.1. from page 51 is reproduced below. The promoter shows relatively high activity in large projecting neurons such as cortical pyramidal cells, but also CA1 and CA3 neurons in the hippocampus. Significant expression levels are also detected in

the hilus. We have chosen this mouse based on the IGFBP5 overexpression pattern that has been reported by Barucker et al., J Alzheimers Dis. 2015;44(2):613-24, because it matches this overexpression.

Brain structure/ core region	Cre expression
**Forebrain**	
Olfactory bulb	+
Piriform cortex	+++
Claustrum	+++
Septum	+
Ammon´s Horn	+++
Gyrus dentatus (granule cells)	-
Gyrus dentatus (hilus)	++
Amygdala	++
Cerebral cortex (pyramidal cells)	++ - +++
Striatum	-
**Diencephalon**	
Thalamus	++
Hypothalamus	-
Locus coeruleus	-
Nucleus ruber	-
Substantia nigra	-
**Brainstem**	
Bridge core	+
Oculomotor nucleus	++
Vestibular nucleus	-
Ventral cochlear nucleus	++
Mesencephalic trigeminal nucleus	++
Trigeminal motor nucleus	+++
Abducens nucleus	-
Facial nerve nucleus	+++
Ambiguous nucleus	++
Hypoglossal nucleus	+++
Cerebellum	
Purkinje cells	+
Granule cells	-
**Spinal cord**	
Motorneurons	++
Interneurons	-
DRGs	++

Summary of NFL-Cre expression in brain and spinal cord. (-) no expression, (+) weak expression < 30%, (++) expression up to 60%, (+++) strong expression > 60% of cells within the structure. Taken from Schweizer Ulrich, Genetische Untersuchungen zur Rolle von Cytochrom C und Stat3 bei der Regulation des embryonalen Zelltods von Motoneuronen der Maus, Dissertation, 2002 Julius-Maximilians-Universität Würzburg, Table 3.2.1, p.51, translated from German into English.

Morris water maze - Calculation of time in the platform area

The target area refers to the area of the platform with 10 cm diameter which is only a small part of the target quadrant (0.38 m^2). Calculation: Time spent in platform area (%) = Spent time (seconds) in platform area x 100 / Spent time (seconds) in target quadrant.

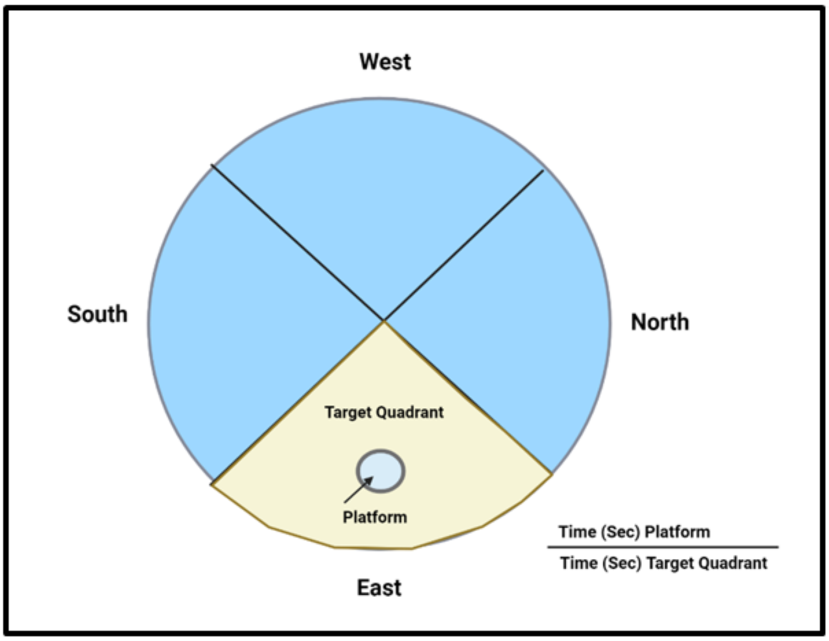

Online Resource Supplementary Figures

Online Resource Fig.1

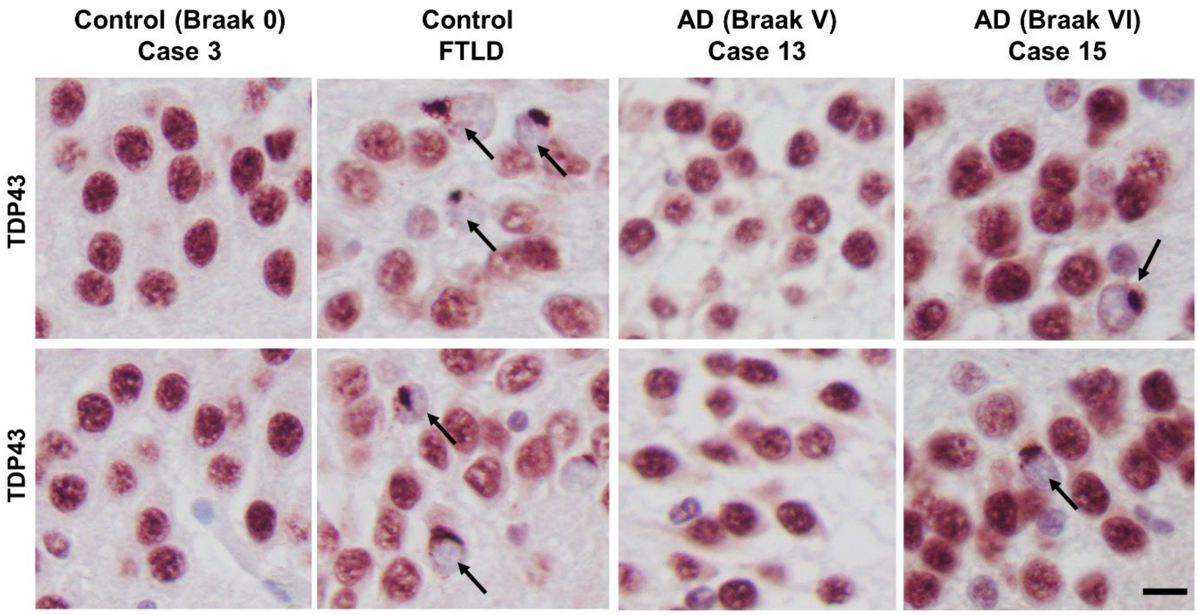

Online Resource Fig.1 TAR-DNA-binding protein 43 (TDP-43) pathology in Alzheimer disease (AD). Immunohistochemistry with anti-TDP-43 showing neuronal cytoplasmic inclusions (arrow) in dentate granule cells in a case with frontal lobe dementia (FTLD) and in an AD case (case 15, Braak VI). Note that AD case 13 (Braak V) showed no neuronal cytoplasmic inclusions in dentate granule cells. Scale bar = 50 µm.

Online Resource Fig.2

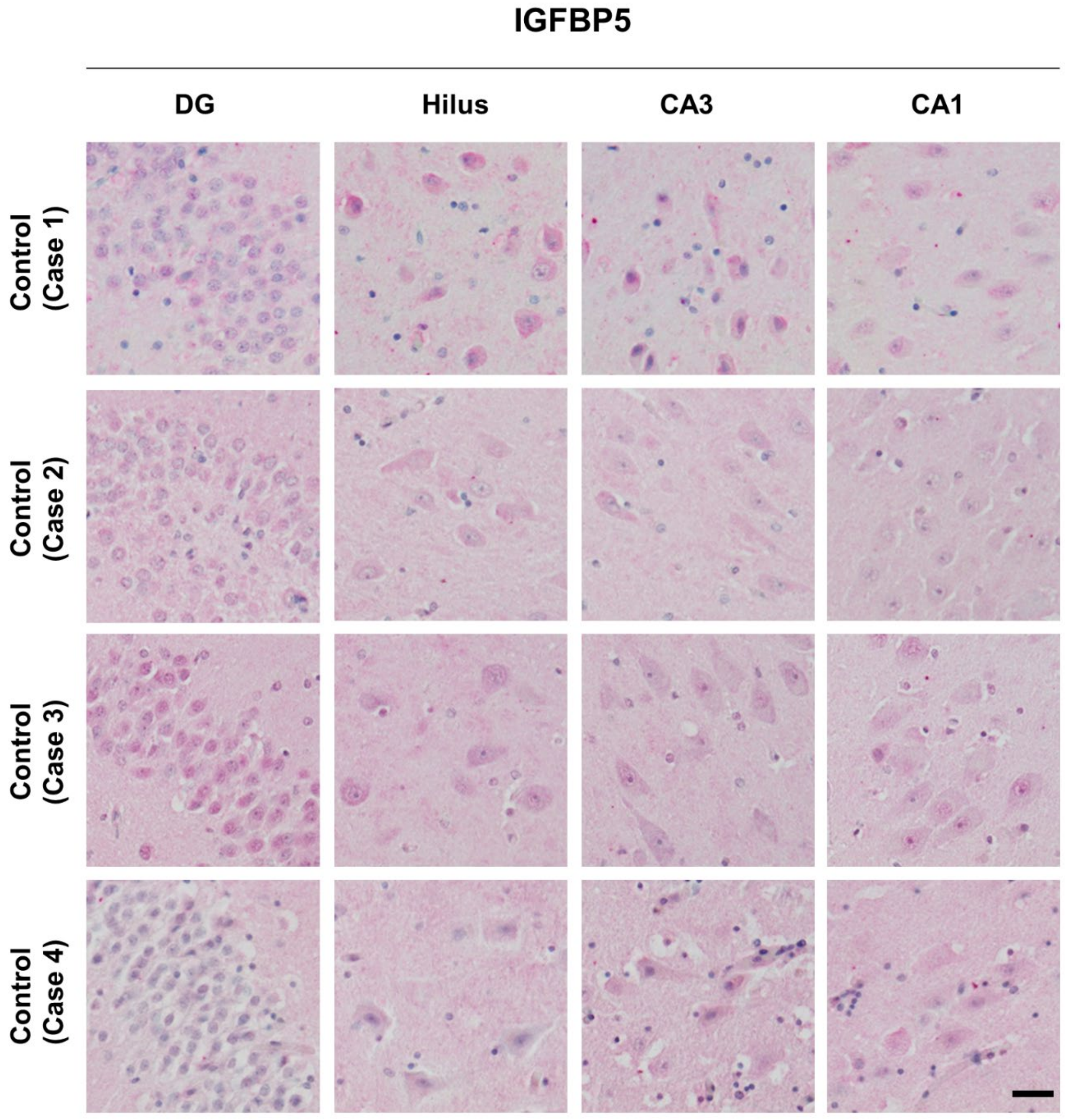

Online Resource Fig.2 IGFBP5 accumulates in hippocampal neurons of Alzheimer cases (AD) compared to control cases. IGFBP5-IR visualized by permanent AP red immunohistochemistry in granule cells of the dentate gyrus, neuronal cells of the hilus, CA3 and CA1 hippocampal fields of all cases listed in Table 1. CA, cornu ammonis; DG, dentate gyrus. Scale bar = 50 µm.

Online Resource Fig.2

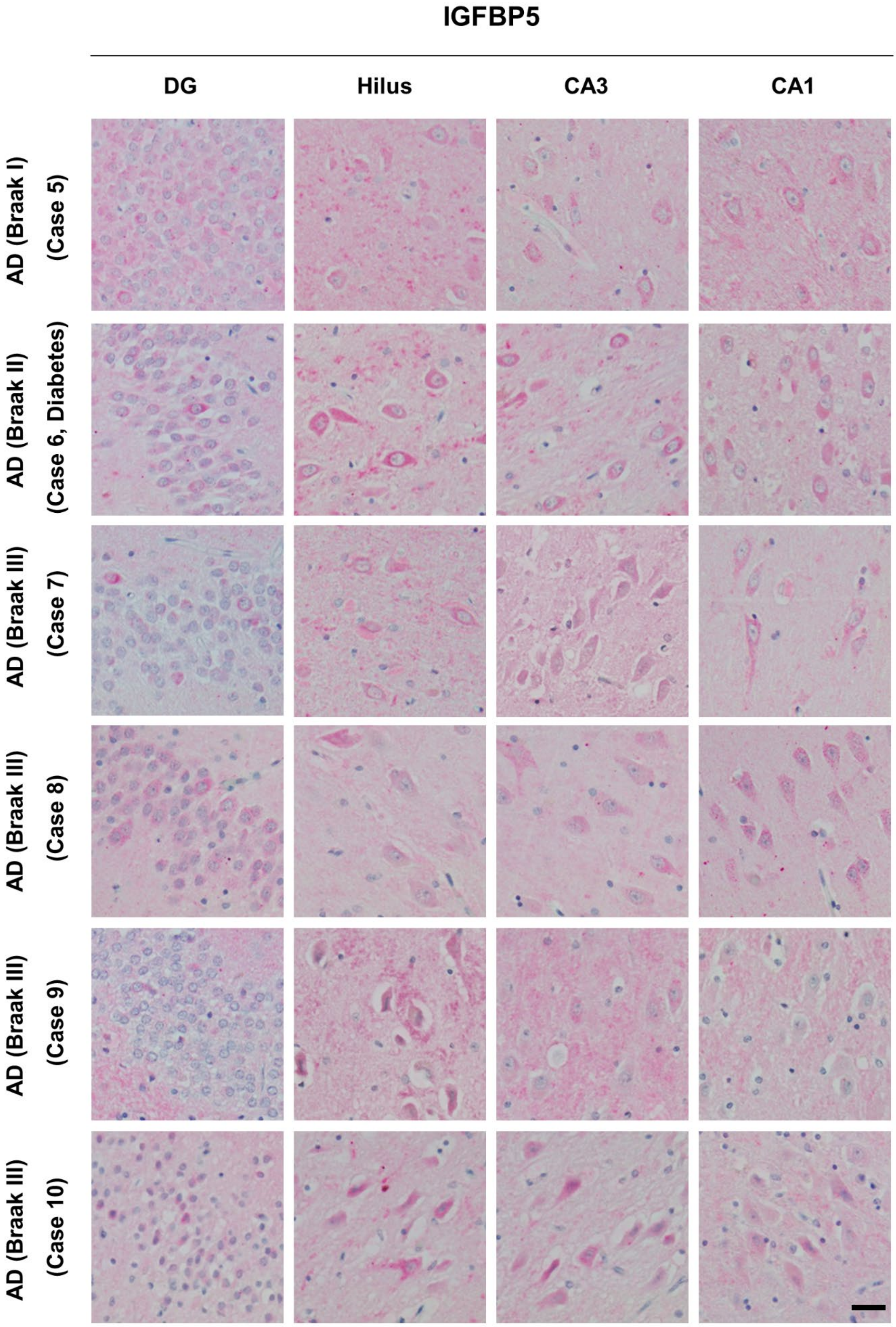

Online Resource Fig.2

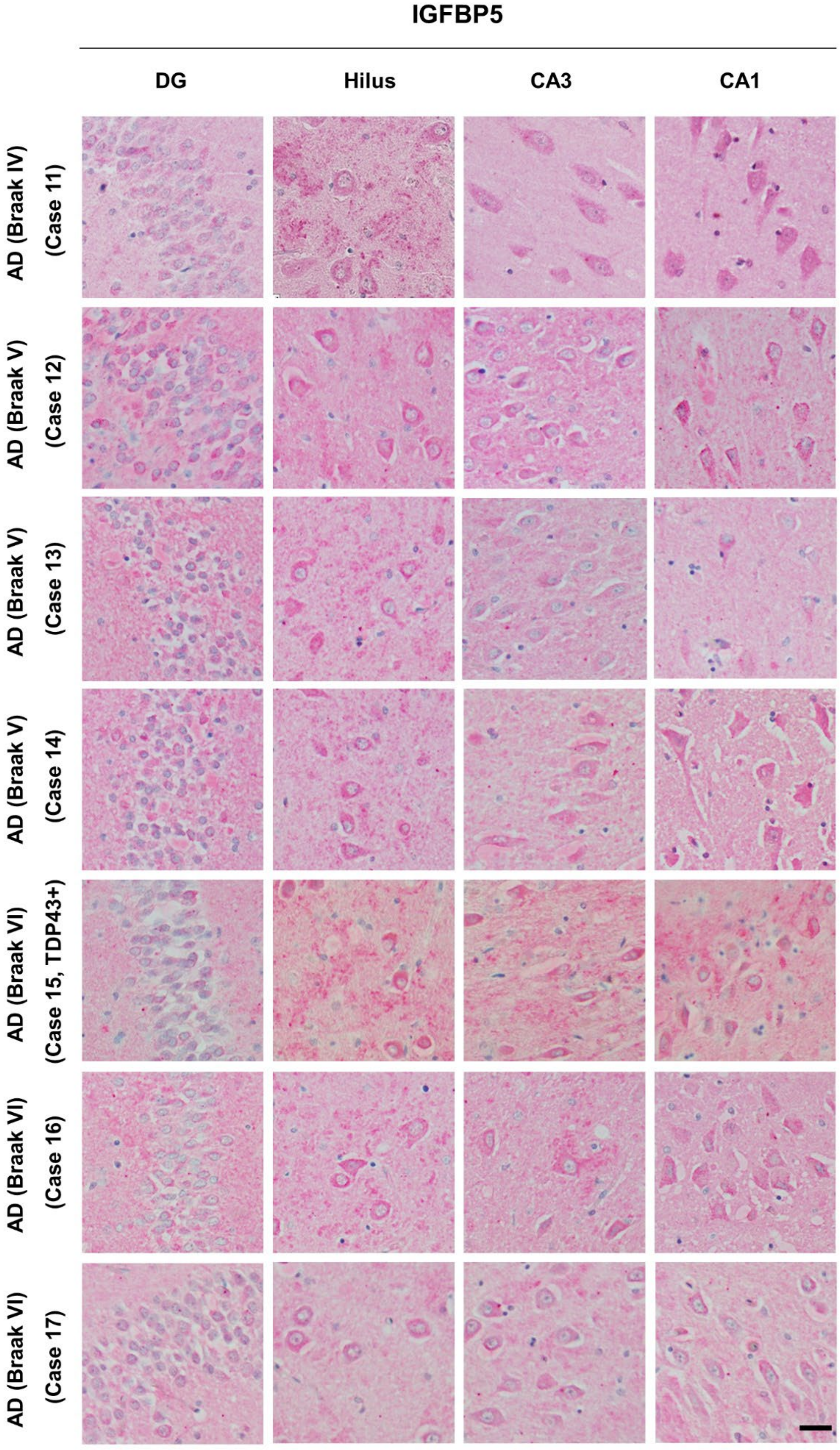

Online Resource Fig.3

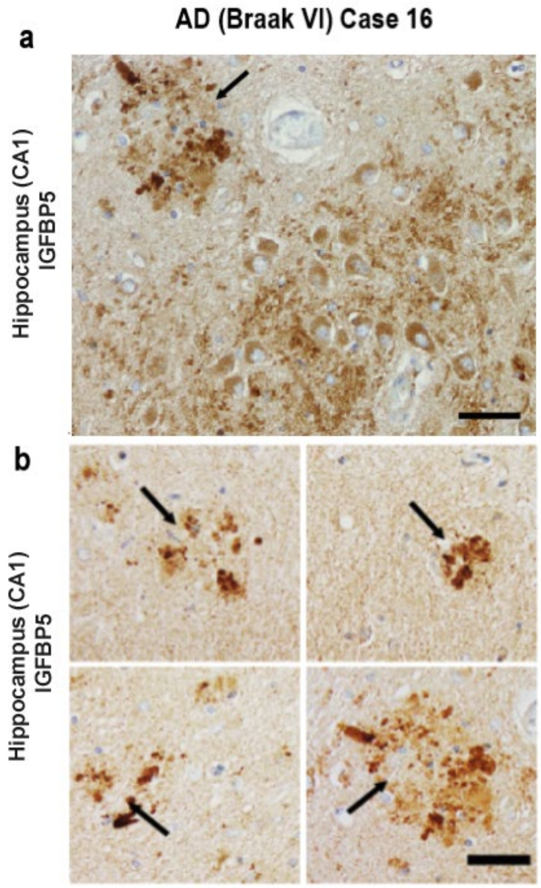

Online Resource Fig.3 IGFBP5 accumulates in hippocampal neurons and around plaques of Alzheimer cases (AD). IGFBP5-IR visualized by DAB immunohistochemistry in CA1 pyramidal neurons and around plaques (arrow) of AD case 16 (Braak VI). Scale bars a = 200 μm, b= 50 μm.

Online Resource Fig.4

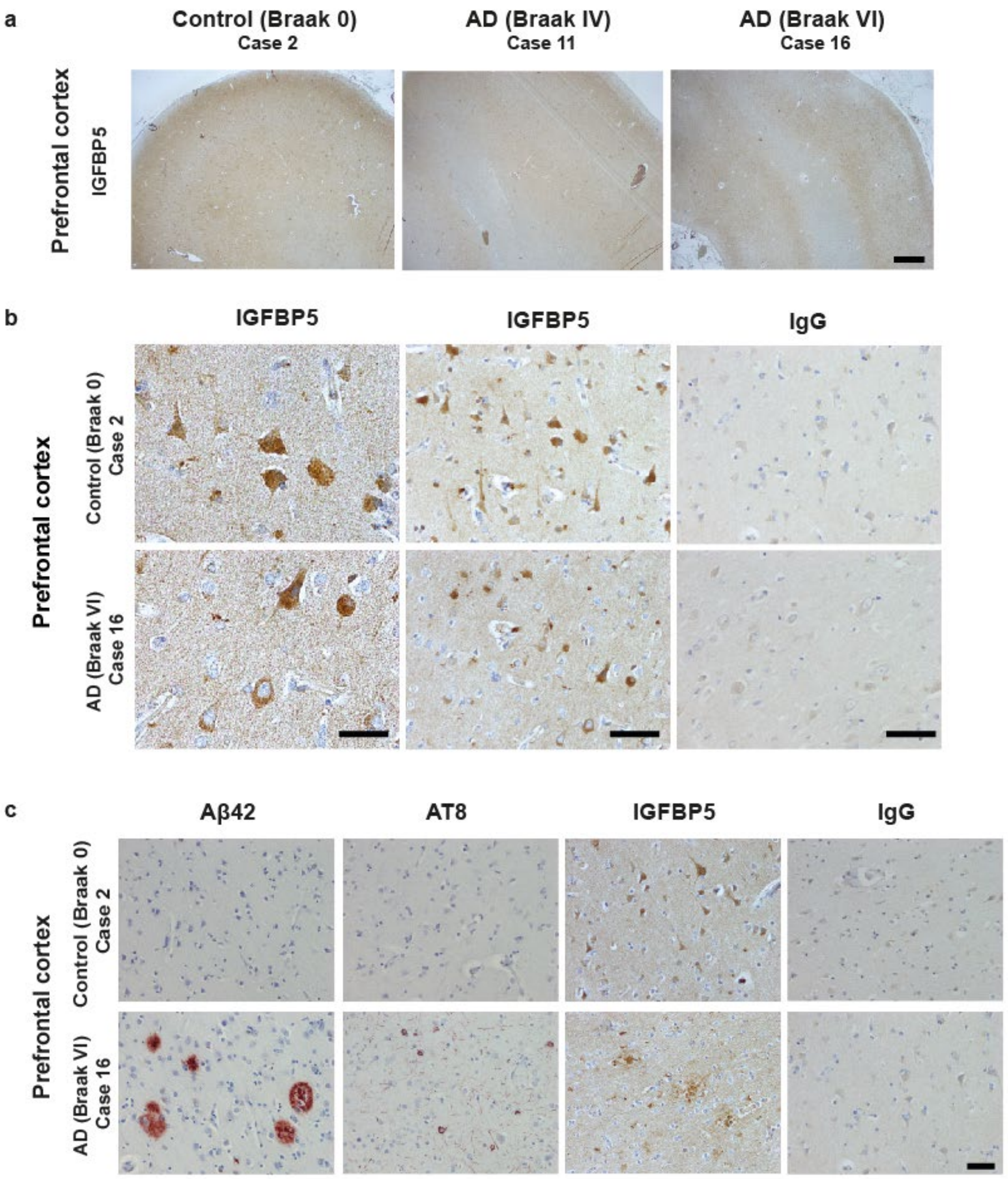

Online Resource Fig.4 In the prefrontal cortex, IGFBP5 does not accumulate in pyramidal neurons and around plaques of Alzheimer cases (AD). a IGFBP5-IR in the prefrontal cortex of control case 2 and AD cases 11 (Braak IV) and 16 (Braak VI) visualized by DAB immunohistochemistry. **b** IGFBP5-IR in the prefrontal cortex was comparable between control case 2 and AD case 16. **c** Representative image of Aß42, AT8, IGFBP5 and IgG control DAB immunostaining in the prefrontal cortex of control case 2 and AD case 16. Scale bar a = 200 µm, b = 50 µm (left picture) 200 µm (middle and right image), c = 50 µm.

Online Resource Fig.5

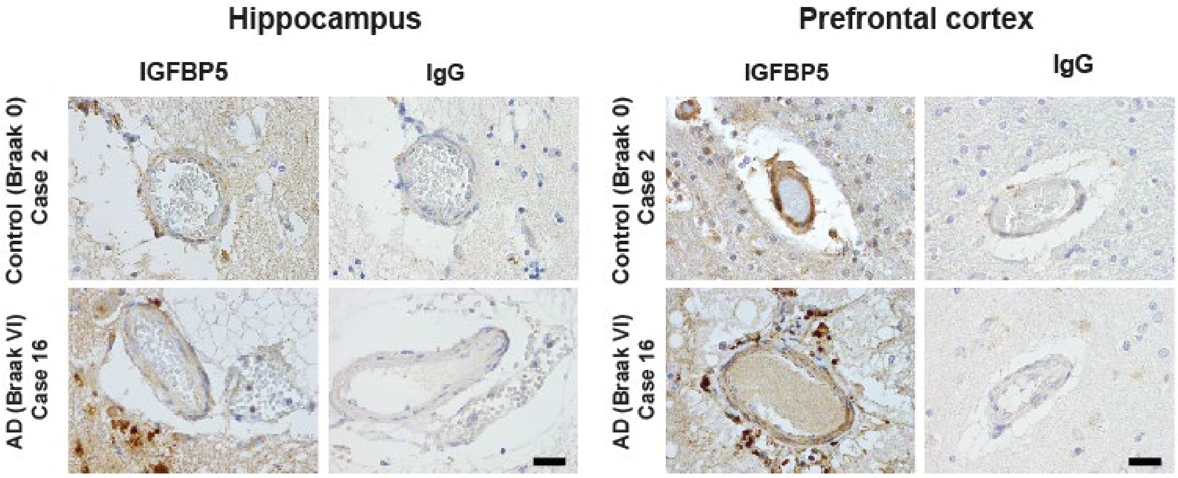

Online Resource Fig.5 Representative images of blood vessels of control case 2 (Braak 0) and AD case 16 (Braak VI) in the hippocampus and prefrontal cortex stained for IGFBP5. IgG, Immunoglobulin. Scale bar 50 μm.

Online Resource Fig.6

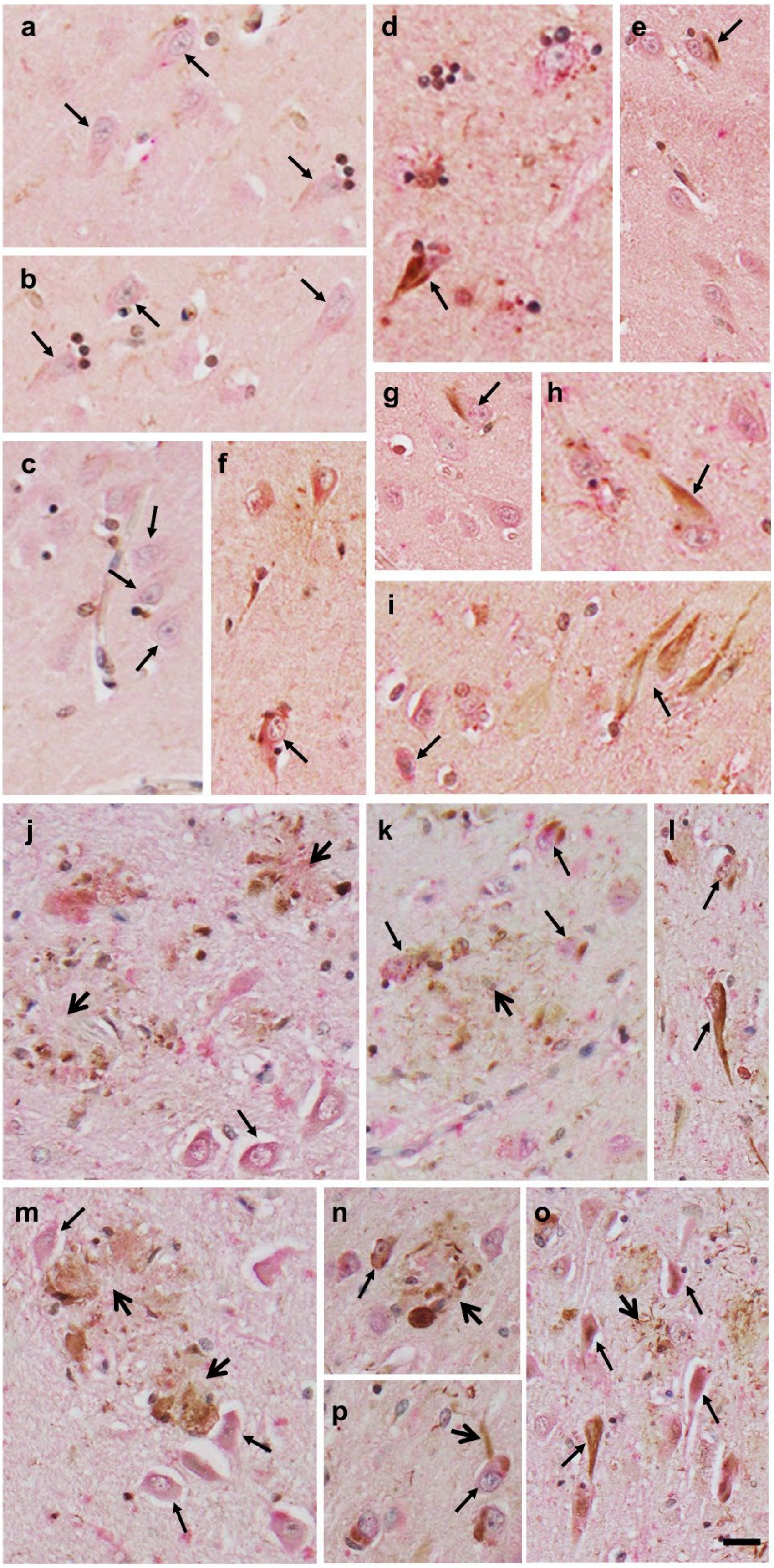

Online Resource Fig.6 Light microscopy of IGFBP5 (red) and ubiquitin (brown) immunoreactivity of CA1 pyramidal neurons, visualized by the chromogenic IHC double staining technique on sections of control and Alzheimer cases. a b c Hippocampal CA1 region of case 2. Weak IGFBP5 immunoreactivity can be visualized in CA1 pyramidal neurons. No ubiquitin positive tangles and plaques are detected. **d e f g h i** Hippocampal CA1 region in cases 6, 8, and 9 constituting representatives of Braak stages I–III. IGFBP5-Ubiquitin positive flame-like neurofibrillary tangles can be identified. **j k l m n o** Hippocampal CA1 region in cases 12, 13, 15 and 16 representatives of Braak stages IV–VI. Strong IGFBP5 positive neurons are found near to ubiquitin positive plaques and tangles. Scale bar = 50 µm.

Online Resource Fig.7

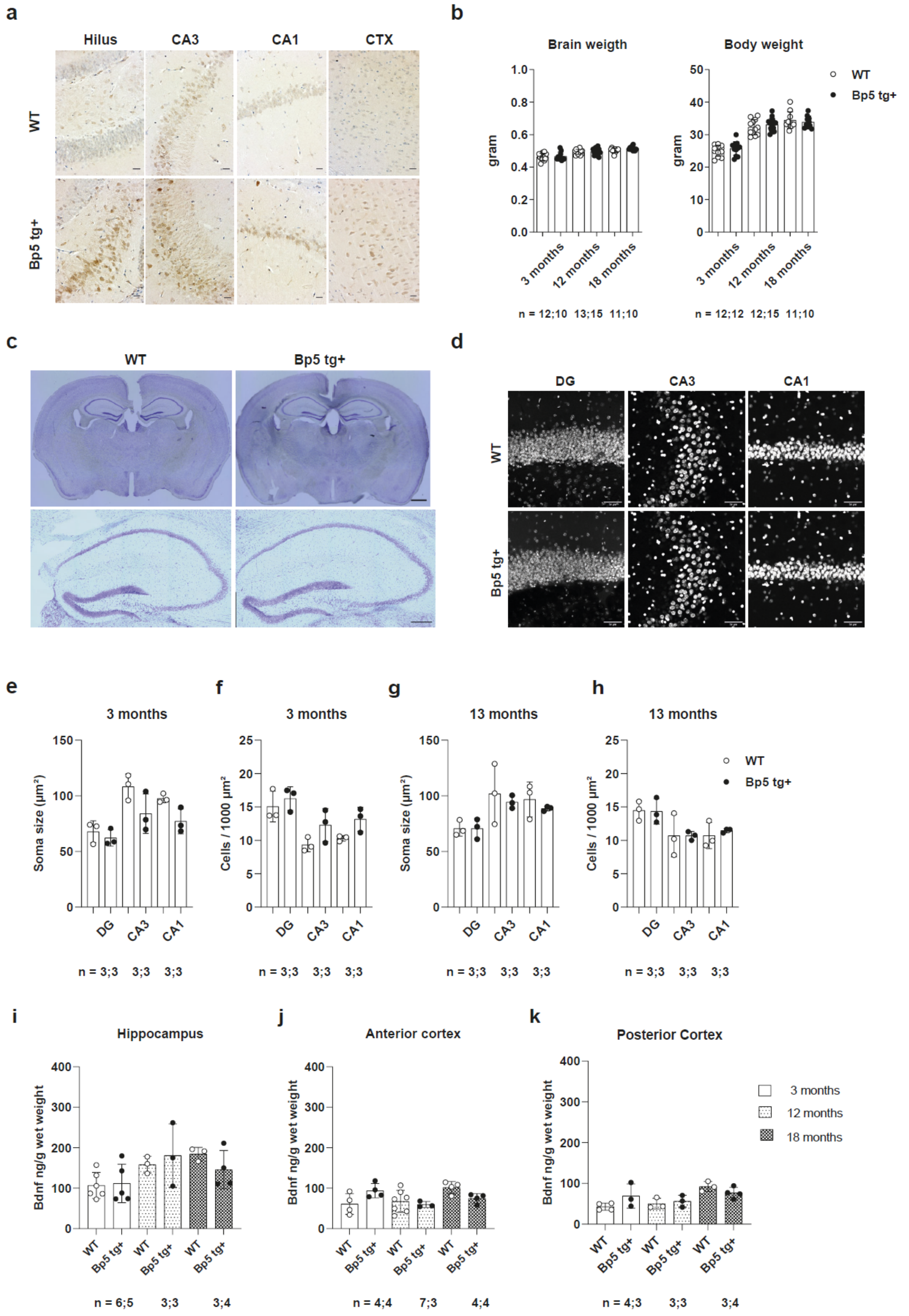

Online Resource Fig.7 Increased Igfbp5 expression has no significant impact on the gross brain morphology and endogenous Bdnf protein levels. a Enhanced Igfbp5-IR in neuronal cell bodies of the hilus, CA3, and CA1 regions of the hippocampus and in cortical layers II/III and V of *Bp5 tg+* mice compared to wild-type mice. **b** Wet weights of total brain as well as body weights showed no significant differences between 3-, 12- and 18-month-old wild-type mice and between age-matched wild-type and *Bp5 tg+* mice**.** Data represent mean ± SD, One-way ANOVA, Tukey's *post hoc* test: *$p < 0.05$. n = number of mice. **c** Cresyl violet staining of coronal brain sections of 3-month-old wild-type and *Bp5 tg+* mice. **d** DAPI stained brain sections showing hippocampal regions of DG, CA3 and CA1 of 3- and 13-month-old wild-type and *Bp5 tg+* mice. **e-h** Soma size and cell density of the DG, CA1 and CA3 hippocampal regions of 3- and 13-month-old wild-type and *Bp5 tg+* mice. Data represent mean ± SD, One-way ANOVA, Tukey's *post hoc* test: *$p < 0.05$. n = number of mice. **i-k** Bdnf protein levels determined by ELISA in the hippocampus, anterior and posterior cortex of 3-, 12-, and 18-month-old wild-type and *Bp5 tg+* mice. Data represent mean ± SD, One-way ANOVA, Tukey's *post hoc* test: *$p < 0.05$. n = number of mice. DAPI (4′, 6'-diamidine-2′-phenylindoledihydro-chloride), dentate gyrus (DG), cornu ammonis 3 (CA3), cornu ammonis1 (CA1); c = Scale bar = coronal brain section 2000 µm, hippocampus 500 µm d = Scale bar = 50 µm.

Online Resource Fig.8

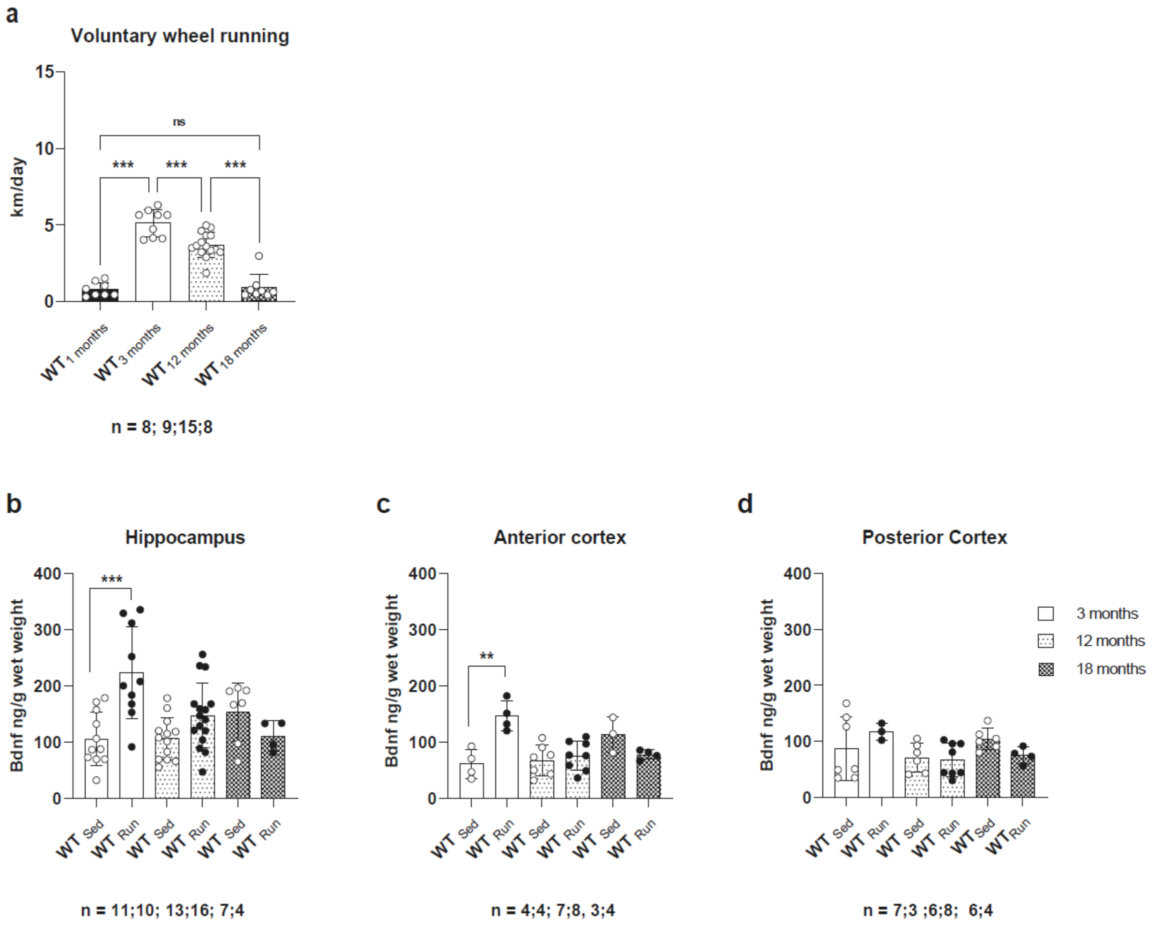

Online Resource Fig.8 Exercise-driven increases of Bdnf protein levels in young but not in aged exercising wild-type mice. a Distance voluntarily completed during the day decreased significantly between 3-, 12-, and 18-month-old wild-type mice. Data represent mean ± SD, One-way ANOVA, Tukey's *post hoc* test: ***$p < 0.001$. n = number of mice. **b-d** Bdnf protein levels determined by ELISA in exercising aging wild-type mice. Data represent mean ± SD, One-way ANOVA, Tukey's *post hoc* test: **$p < 0.01$, ***$p < 0.001$. n = number of mice.

Online Resource Fig.9

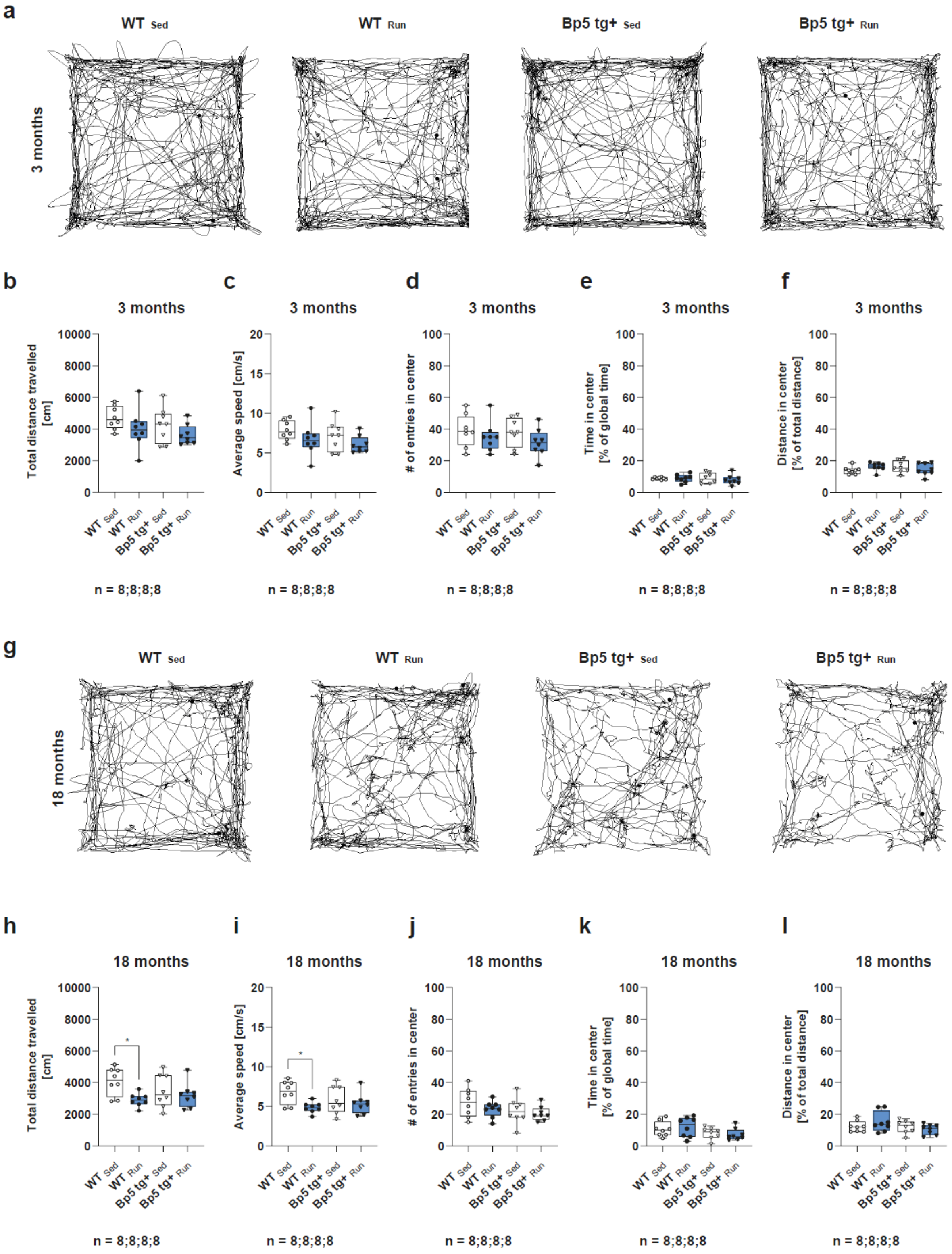

Online Resource Fig.9 Increased neuronal Igfbp5 expression and voluntary exercise do not significantly affect general motor activity, exploratory- or anxiety-like behaviour in 3- and 18-month-old mice. **a** Representative traces showing the movement of 3-month-old sedentary wild-type (WT_{Sed}), running wild-type (WT_{Run}), sedentary *Bp5 tg+* (*Bp5 tg+*$_{Sed}$) and running *Bp5 tg+(Bp5 tg+*$_{Run}$) mice in the open field arena during 10 minutes after being placed in the open field. **b** Total distance travelled by 3-month-old sedentary wild-type (WT_{Sed}), running wild-type (WT_{Run}), sedentary *Bp5 tg+* (*Bp5 tg+*$_{Sed}$) and running *Bp5 tg+(Bp5 tg+*$_{Run}$) mice in the open field arena. **c** Average speed of 3-month-old sedentary wild-type (WT_{Sed}), running wild-type (WT_{Run}), sedentary *Bp5 tg+* (*Bp5 tg+*$_{Sed}$) and running *Bp5 tg+(Bp5 tg+*$_{Run}$) mice in the open field arena. **d** Number of entries in the center area by 3-month-old sedentary wild-type (WT_{Sed}), running wild-type (WT_{Run}), sedentary *Bp5 tg+* (*Bp5 tg+*$_{Sed}$) and running *Bp5 tg+(Bp5 tg+*$_{Run}$) mice in the open field arena **e** Time spent in center (% of global time) by 3-month-old sedentary wild-type (WT_{Sed}), running wild-type (WT_{Run}), sedentary *Bp5 tg+* (*Bp5 tg+*$_{Sed}$) and running *Bp5 tg+(Bp5 tg+*$_{Run}$) mice in the open field arena. **f** Distance in center (% of total distance) of 3-month-old sedentary wild-type (WT_{Sed}), running wild-type (WT_{Run}), sedentary *Bp5 tg+* (*Bp5 tg+*$_{Sed}$) and running *Bp5 tg+(Bp5 tg+*$_{Run}$) mice in the open field arena. **g** Representative traces showing the movement of 18-month-old sedentary wild-type (WT_{Sed}), running wild-type (WT_{Run}), sedentary *Bp5 tg+* (*Bp5 tg+*$_{Sed}$) and running *Bp5 tg+(Bp5 tg+*$_{Run}$) mice in the open field arena during 10 minutes after being placed in the open field. **h** Total distance travelled by 18-month-old sedentary wild-type (WT_{Sed}), running wild-type (WT_{Run}), sedentary *Bp5 tg+* (*Bp5 tg+*$_{Sed}$) and running *Bp5 tg+(Bp5 tg+*$_{Run}$) mice in the open field arena. **i** Average speed of 18-month-old sedentary wild-type (WT_{Sed}), running wild-type (WT_{Run}), sedentary *Bp5 tg+* (*Bp5 tg+*$_{Sed}$) and running *Bp5 tg+(Bp5 tg+*$_{Run}$) mice in the open field arena. **j** Number of entries in center by 18-month-old sedentary wild-type (WT_{Sed}), running wild-type (WT_{Run}), sedentary *Bp5 tg+* (*Bp5 tg+*$_{Sed}$) and running *Bp5 tg+(Bp5 tg+*$_{Run}$) mice in the open field arena. **k** Time in center (% of global time) by 18-month-old sedentary wild-type (WT_{Sed}), running wild-type (WT_{Run}), sedentary *Bp5 tg+* (*Bp5 tg+*$_{Sed}$) and running *Bp5 tg+(Bp5 tg+*$_{Run}$) mice in the open field arena. **l** Distance in center (% of total distance) by 18-month-old sedentary wild-type (WT_{Sed}), running wild-type (WT_{Run}), sedentary *Bp5 tg+* (*Bp5 tg+*$_{Sed}$) and running *Bp5 tg+(Bp5 tg+*$_{Run}$) mice in the open field arena. Data represent mean ± SD, One-way ANOVA, Tukey's *post hoc* test: $^*p < 0.05$. n = number of mice.

Online Resource Fig.10

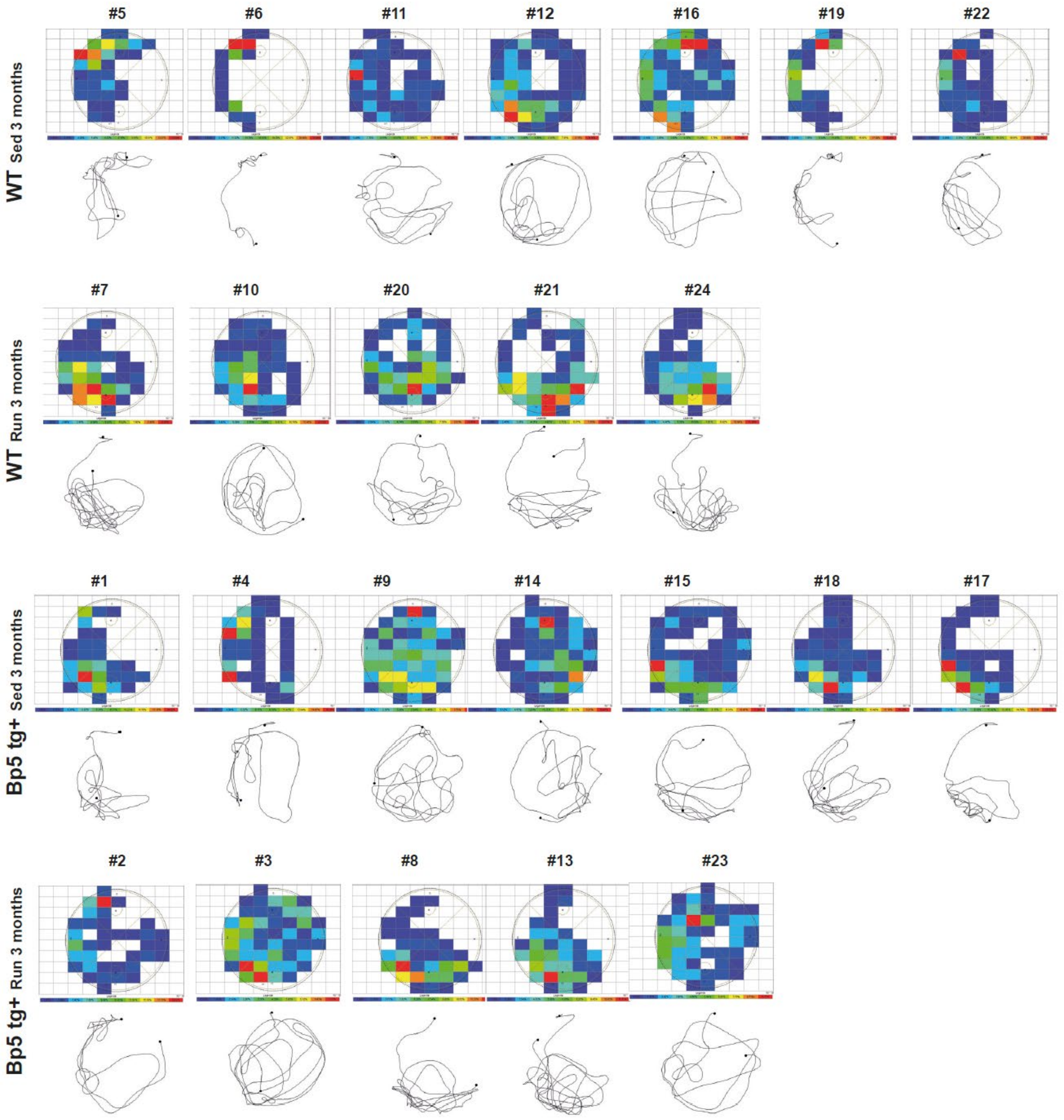

Online Resource Fig.10 Representative traces showing the movement of 3-month-old sedentary wild-type (WT_{Sed}), running wild-type (WT_{Run}), sedentary *Bp5 tg+* (*Bp5 tg+*$_{Sed}$) and running *Bp5 tg+(Bp5 tg+*$_{Run}$) mice in the Morris water maze during the probe trial on day 6.

Online Resource Fig.11

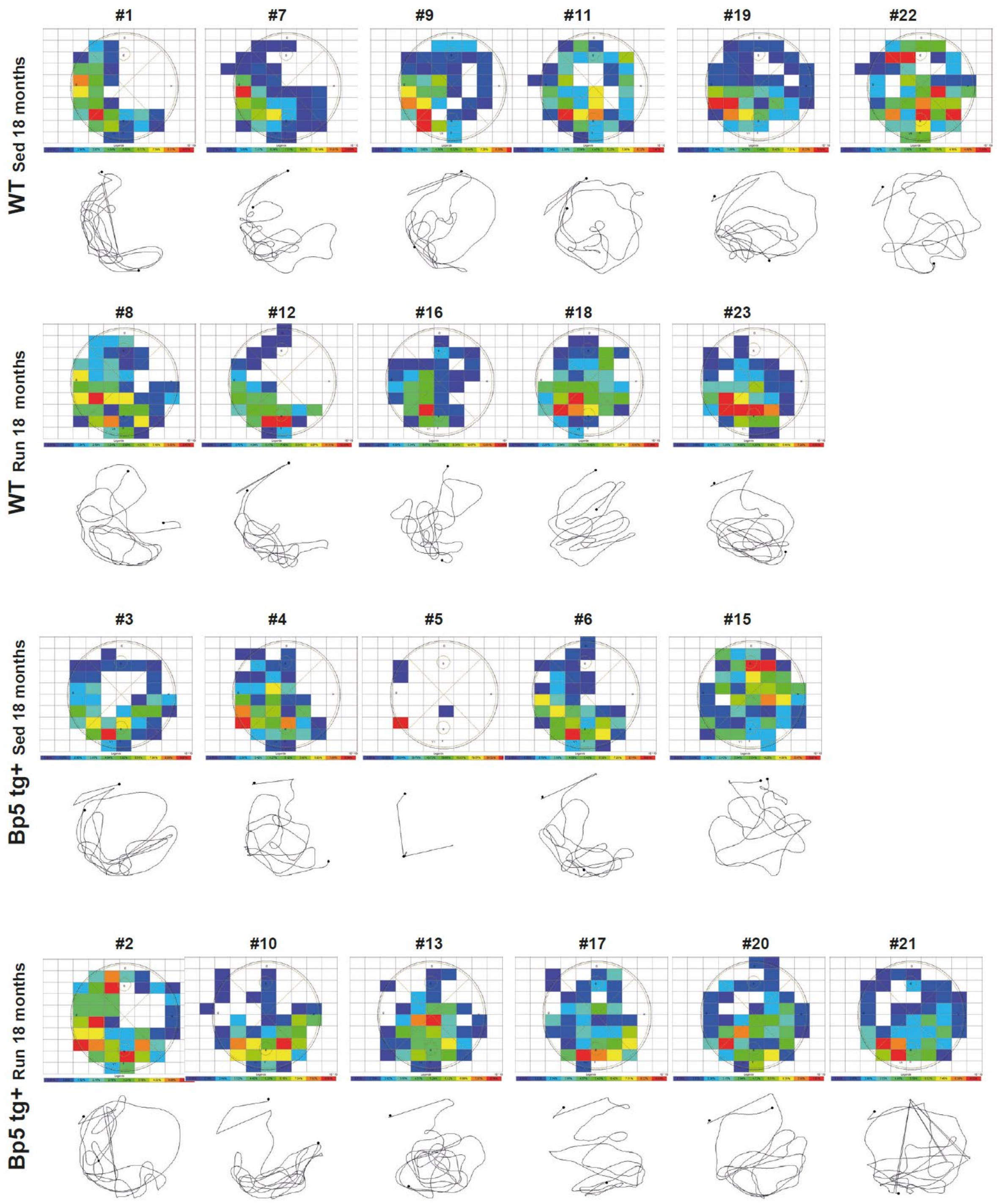

Online Resource Fig.11 Representative traces showing the movement of 18-month-old sedentary wild-type (WT_{Sed}), running wild-type (WT_{Run}), sedentary *Bp5 tg+* (*Bp5 tg+*$_{Sed}$) and running *Bp5 tg+(Bp5 tg+*$_{Run}$) mice in the Morris water maze during the probe trial on day 6.

Online Resource Fig.12

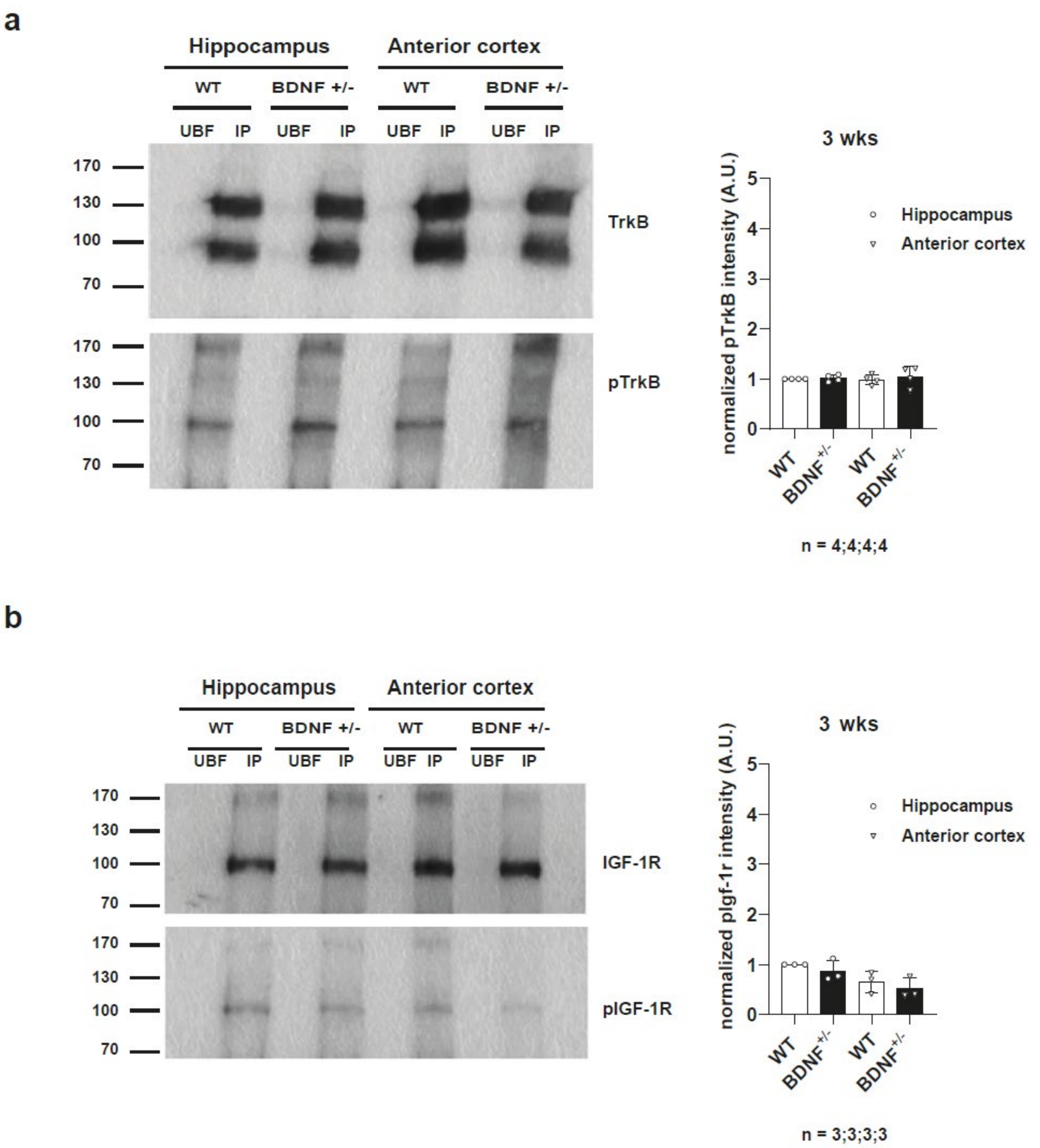

Online Resource Fig.12 TrkB and Igf-1r activation do not correlate with Bdnf protein levels in the brain. a TrkB and **e-h** Igf-1r immunoprecipitation from hippocampal and cortical lysates of 3-week-old wild-type and *Bdnf* heterozygous knockout mice ($Bdnf^{+/-}$) mice. Data represent mean ± SD, ANOVA, Kruskal-Wallis test, Dunn´s *post hoc* test, * $p < 0.05$, n = number of mice. Values were normalized to 3-week-old hippocampus. UBF unbound fraction, IP immunoprecipitation, $BDNF^{+/-}$ = BDNF heterozygous knockout mice.
